# In vitro exposure of a 3D-tetraculture representative for the alveolar barrier at the air-liquid interface to silver particles and nanowires

**DOI:** 10.1186/s12989-019-0297-1

**Published:** 2019-04-02

**Authors:** Ionel Fizeșan, Sébastien Cambier, Elisa Moschini, Aline Chary, Inge Nelissen, Johanna Ziebel, Jean-Nicolas Audinot, Tom Wirtz, Marcin Kruszewski, Anca Pop, Béla Kiss, Tommaso Serchi, Felicia Loghin, Arno C. Gutleb

**Affiliations:** 1 Toxicology Department, Faculty of Pharmacy, Iuliu Hațieganu University of Medicine and Pharmacy, Cluj-Napoca, Romania; 2 Environmental Research and Innovation (ERIN) Department, Luxembourg Institute of Science and Technology, Belvaux, Luxembourg; 3 Health Unit, Flemish Institute for Technological Research (VITO NV), Mol, Belgium; 4 Material Research and Technology (MRT) Department, Luxembourg Institute of Science and Technology, Belvaux, Luxembourg; 5 Faculty of Medicine, University of Information Technology and Management in Rzeszow, Sucharskiego 2, Rzeszow, Poland; 6 Centre for Radiobiology and Biological Dosimetry, Institute of Nuclear Chemistry and Technology, Dorodna 16, Warszawa, Poland

**Keywords:** Air-liquid interface, Tetra-culture, Silver nanoparticles, Silver nanowires, Inflammation;

## Abstract

**Background:**

The present study aimed to evaluate the potential differences in the biological effects of two types of spherical silver particles of 20 and 200 nm (Ag20 and Ag200), and of PVP-coated silver nanowires (AgNWs) with a diameter of 50 nm and length up to 50 μm, using a complex 3D model representative for the alveolar barrier cultured at air-liquid interface (ALI). The alveolar model was exposed to 0.05, 0.5 and 5 μg/cm^2^ of test compounds at ALI using a state-of-the-art exposure system (Vitrocell™Cloud System). Endpoints related to the oxidative stress induction, anti-oxidant defence mechanisms, pro-inflammatory responses and cellular death were selected to evaluate the biocompatibility of silver particles and nanowires (AgNMs) and to further ascribe particular biological effects to the different morphologic properties between the three types of AgNMs evaluated.

**Results:**

Significant cytotoxic effect was observed for all three types of AgNMs at the highest tested doses. The increased mRNA levels of the pro-apoptotic gene *CASP7* suggests that apoptosis may occur after exposure to AgNWs. All three types of AgNMs increased the mRNA level of the anti-oxidant enzyme *HMOX-1* and of the metal-binding anti-oxidant *metallothioneins* (*MTs*), with AgNWs being the most potent inducer. Even though all types of AgNMs induced the nuclear translocation of NF-kB, only AgNWs increased the mRNA level of pro-inflammatory mediators. The pro-inflammatory response elicited by AgNWs was further confirmed by the increased secretion of the 10 evaluated interleukins.

**Conclusion:**

In the current study, we demonstrated that the direct exposure of a complex tetra-culture alveolar model to different types of AgNMs at ALI induces shape- and size-specific biological responses. From the three AgNMs tested, AgNWs were the most potent in inducing biological alterations. Starting from 50 ng/cm^2^, a dose representative for an acute exposure in a high exposure occupational setting, AgNWs induced prominent changes indicative for a pro-inflammatory response. Even though the acute responses towards a dose representative for a full-lifetime exposure were also evaluated, chronic exposure scenarios at low dose are still unquestionably needed to reveal the human health impact of AgNMs during realistic conditions.

**Electronic supplementary material:**

The online version of this article (10.1186/s12989-019-0297-1) contains supplementary material, which is available to authorized users.

## Background

The rapid advancements in the field of nanotechnology produced a vast number of engineered nanomaterials (ENMs) with unique physical and chemical properties. Exhibiting a broad utility and versatility, ENMs are currently incorporated in a large number of consumer products, offering also unique advantages as diagnostic and therapeutic tools in the emerging field of nanomedicine [1]. Silver nanomaterials (AgNMs) are one of the most widely used ENMs, with more than 300 commercially available products in Europe [2], due to their antimicrobial activity [3, 4]. Silver nanowires (AgNWs) are incorporated in flexible thin films with high conductivity, and further used in the production of liquid crystal displays, touch screens and solar cells [5, 6].

Respiratory exposure is of primary concern due to release of AgNMs to the ambient air during manufacturing processes and the use of consumer products. Inhaled ENMs are efficiently deposited given the high intake of air (> 20 m^3^/day) and the large surface area (75–150 m^2^) of the respiratory tract. The alveolar barrier is of main concern due to the deposition of small particles and the susceptibility of the gas exchange membrane [7, 8].

Despite the pulmonary retention and systemic distribution of spherical AgNMs, the majority of in vivo studies reported no or only mild effects involving chronic pulmonary inflammation and abnormalities in the liver [9–12]. In contrast, pronounced effects including cytotoxicity, pro-inflammatory responses and genotoxicity were reported using various epithelial and immune cell types in in vitro studies [13–18]. These differences between in vivo and in vitro data are most likely due to the unrealistic exposure scenarios and concentrations used. Submerged exposure of alveolar cells has several intrinsic limitations, as it disregards the basic structure of the respiratory system and the physical laws behind respiratory exposure. Moreover, by suspending ENMs in cell culture media, important changes in their physicochemical properties including surface reactivity and agglomeration state occur influencing the exposure concentrations and outcome of the experiments [19, 20]. An improved in vitro technique which encompasses the above-mentioned limitations is represented by air-liquid interface (ALI) exposure [21]. This allows a direct exposure to ENMs, closely mimicking realistic inhalation conditions [19, 22–24]. It is generally accepted that the use of ALI exposure in respiratory toxicology is more suitable in the assessment of ENMs toxicity, and together with other improved in vitro approaches may hold the promise to bridge the current gaps between in vitro and in vivo data [19, 25].

The aim of the present study was to assess and compare the toxicological effects of two types of spherical silver particles with sizes of 20 and 200 nm (Ag20 and Ag200, respectively) and of AgNWs with a diameter of 50 nm and a length up to 50 μm, using a 3D tetra-culture model representative for the alveolar barrier [22, 26, 27]. The model was exposed to 0.05, 0.5 and 5 μg/cm^2^ of AgNMs at the ALI using a state-of-the-art exposure system (Vitrocell™Cloud System). Specific endpoints related to the oxidative stress induction, anti-oxidant defence mechanisms, pro-inflammatory responses and cellular death were evaluated at different time points.

## Results

### Characterization of ENMs in liquid suspension

Based on the rationale that spraying a homogenous suspension is a prerequisite for a homogenous exposure, the first step was the characterization of the AgNMs in liquid suspension. Preparation of the AgNMs dispersions according to a published protocol [20] resulted in a mean hydrodynamic diameter of 232 ± 125 nm, 362 ± 56 nm and 262 ± 160 nm for Ag20, Ag200 and AgNWs. The measured Zeta potentials for Ag20, Ag200 and AgNWs were − 40.5 ± 4.8, − 32.2 ± 4.6 and − 24.6 ± 8.2 mV, respectively. The values obtained for AgNWs served to calculate the necessary energy input to obtain a stable suspension and are not an accurate measurement of the hydrodynamic diameter, as the Dynamic Light Scattering (DLS) method is based on the assumption of a spherical shape [28, 29]. The stability of AgNMs suspension was not verified at later time points, as suspensions were prepared freshly before each exposure. According to BET measurement, the specific surface area of the Ag20 and Ag200 were 2.52 ± 0.05 and 1.18 ± 0.01 m^2^/g, while the specific surface area for the AgNWs was not measured due to specificities of these AgNWs.

Based on the Inductively Coupled Plasma Mass Spectrometry (ICP-MS) analysis, we observed that the sonication process had a minor impact on the release of Ag^+^ from all AgNMs tested, with Ag^+^ representing less than 0.2% from total silver species immediately after the sonication process. The stability of the AgNMs suspensions was maintained at the same Ag^+^ level over 24 h. Furthermore, incubation with the pulmonary surfactant did not increase the release of Ag^+^ for either of all AgNMs tested.

### Characterization of the deposited ENMs

**Fig. 1:**
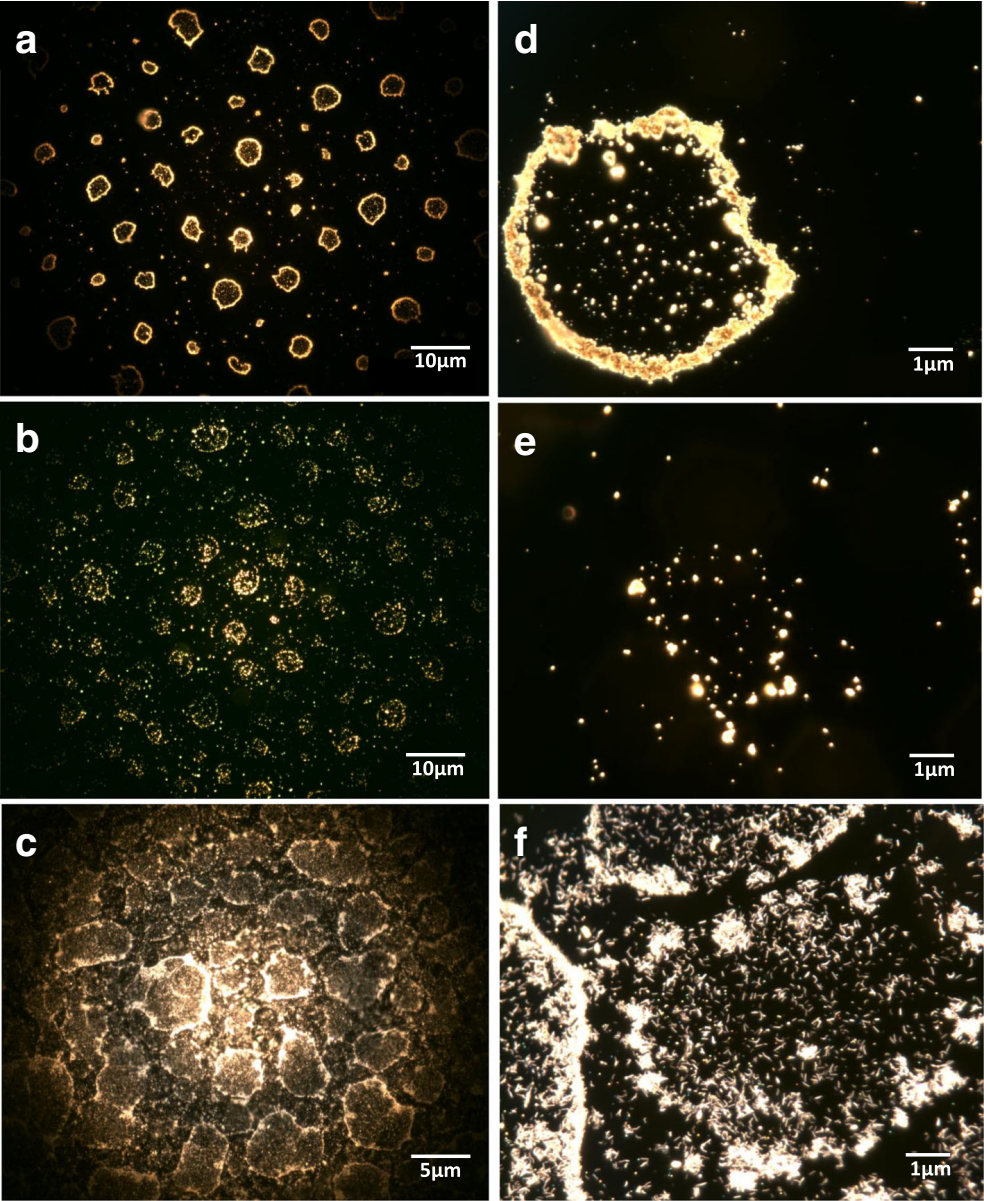
High resolution dark-field microscopy images (CytoViva®) of Ag20 (**a**, **d**), Ag200 (**b**, **e**) and AgNWs (**c**, **f**) after sonication and nebulisation on coverslips using the Vitrocell™Cloud System. Sampl*e*s were further fixed between two coverslips using Mowiol mounting media

**Fig. 2:**
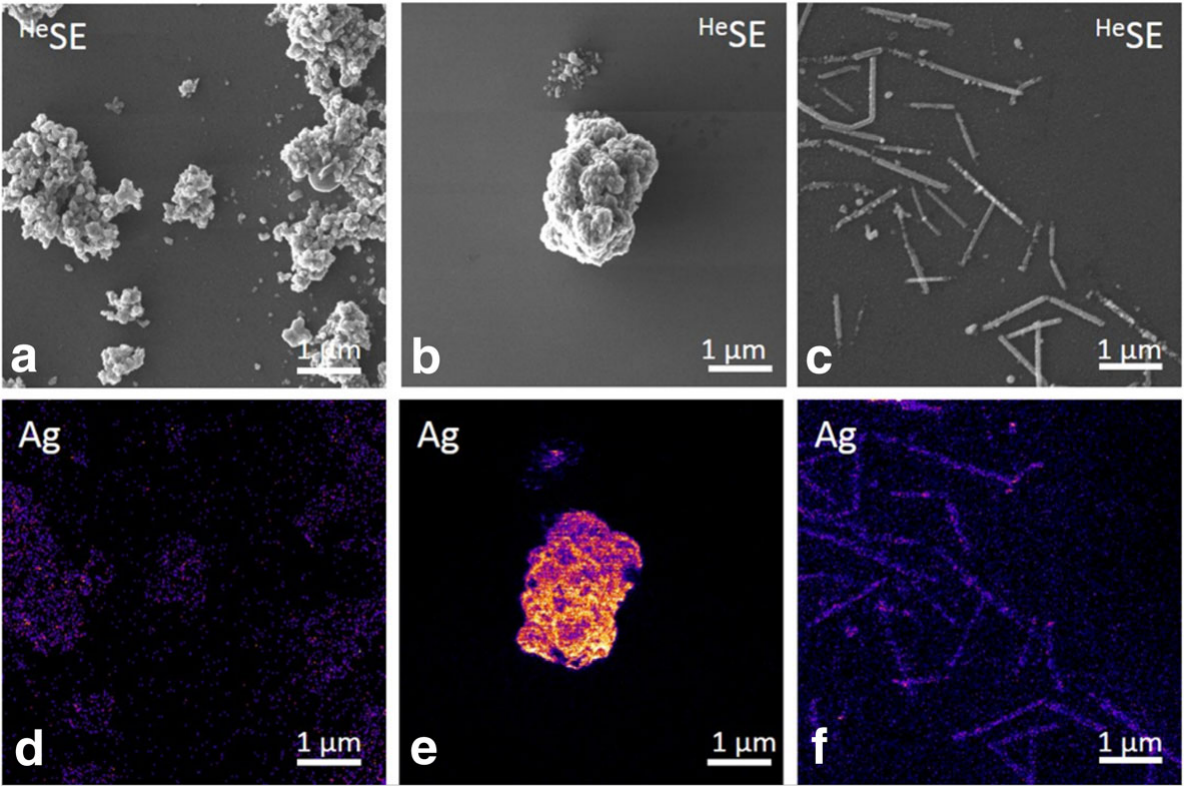
Secondary electron images (**a**-**c**) and mass filtered secondary ion images (**d**-**f**) of Ag20, Ag200 and AgNWs (bar scale 1 μm) obtained on the Helium Ion Microscopy – Secondary Ion Mass Spectrometry (HIM-SIMS) instrument. Samples were deposited on a Silicon wafer

The deposition uniformity was evaluated by two visualization techniques. Using the high-resolution dark-field microscopy technique (CytoViva®), a circular-like deposition pattern rather than a homogenous particle distribution was observed with the majority of AgNMs distributed on the exterior of the circle (Fig. 1). This pattern is in accordance with the droplet size distribution generated by the nebulizer, and may be due to the drying of highly concentrated droplets onto the glass substrate [30, 31]. We believe that this artefact is not present when the cellular model is exposed to the AgNMs due to the presence of the liquid lining. The shape and the analytical composition of the particles were investigated by Helium Ion Microscopy (ZEISS, Peabody, USA) (HIM) [32], coupled with a Secondary Ion Mass Spectrometer (SIMS) developed by LIST, Luxembourg [33, 34]. The sprayed AgNWs maintained their needle-like shape after the sonication and nebulisation processes, with the same ratio between length and diameter being observed as for pristine AgNWs (Fig. 2; Additional file 1: Figure S1). Smaller fragments with more symmetric morphology were also observed (Fig. 2).

Quantification of total silver species using ICP-MS allowed the measurement of the deposition efficiency and the intra- and inter-exposure variability, with the overall validation of the exposure system (Table 1 and Additional file 2: Table S1). For studying the biological effects, 200 μL of AgNMs suspensions with concentrations of approximately 5000, 500 and 50 μg/mL (dependent on the deposition efficiencies obtained previously) were sprayed in order to achieve a deposition of 0.05, 0.5 and 5 μg/cm^2^.

**Table 1:** Deposition efficiencies obtained for Ag20, Ag200 and AgNWs using the Vitrocell™Cloud System

	Concentration of the sprayed suspension (μg/mL)	Deposition efficiency (%)	Expected concentration (μg/cm^2^)	Measured concentration (μg/cm^2^)
Ag20	50	54	0.05	0.054
	500	68	0.5	0.68
	5000	74	5	7.4
Ag200	50	50	0.05	0.05
	500	47	0.5	0.47
	5000	82	5	8.2
AgNWs	50	40	0.05	0.04
	500	57	0.5	0.57
	5000	60	5	6.0

The deposition efficiencies were measured for 3 increasing concentrations

### Cytotoxicity and metabolic impairment

**Fig. 3:**
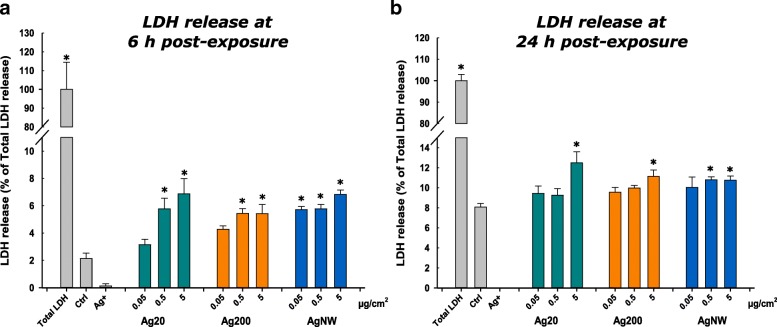
Effects of Ag20, Ag200, AgNWs and Ag^+^ (16 μg/cm^2^) on LDH release at 6 h (**a**) respectively at 24 h post-exposure (**b**). Cells exposed to vehicle served as negative control (Ctrl). Data are presented as the mean ± SD of at least three biological replicates. Data were expressed as the percentage of the LDH release from total LDH release (100%)

The cytotoxic effects after exposure to AgNMs were assessed by measuring the release of lactate dehydrogenase (LDH). All three types of AgNMs tested had a statistically significant impact on the cellular integrity and the membrane leakage of LDH (Fig. 3). The 16 μg/cm^2^ (Ag^+^) silver nitrate solution used as a positive control for toxicity interfered with the accurate measurement of the LDH activity, this interference of Ag^+^ being also reported in other studies [35]. The integrity of the cellular layer after exposure to AgNWs was further evaluated by confocal microscopy. Exposure to the AgNWs at the highest concentration did not reveal any significant effects on the apical compartment of the alveolar model (Additional file 3: Figure S2).

**Fig. 4:**
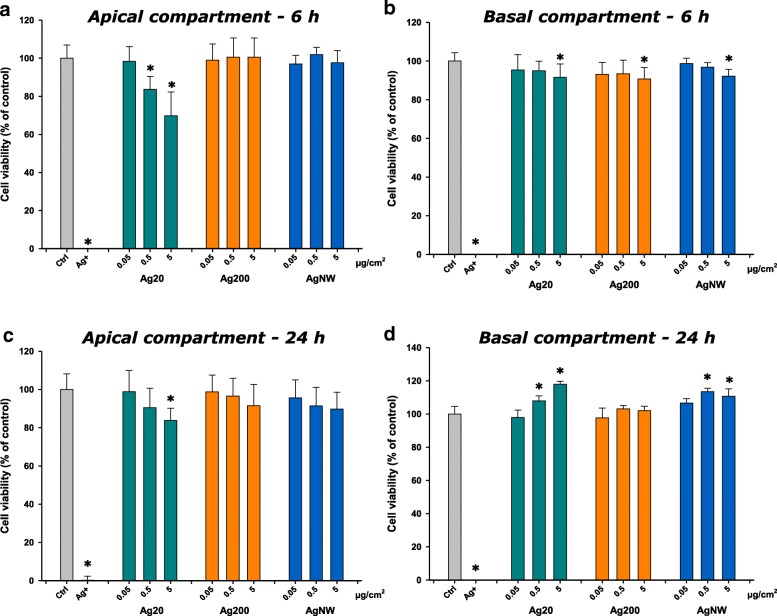
Effects of Ag20, Ag200, AgNWs and Ag^+^(16 μg/cm^2^) on cellular viability using Alamar Blue Assay at 6 h post-exposure on apical (**a**) and basolateral side (**b**), respectively at 24 h post exposure on apical (**c**) and basolateral side (**d**). Cells exposed to vehicle served as negative control (Ctrl). The data are presented as the mean ± SD of at least three biological replicates. Data were expressed relative to the negative control (100%)

The cytotoxic effects elicited by AgNMs were further assessed using the Alamar Blue (AB) assay. Ag20 had a visible dose-dependent effect, decreasing the cellular viability in the apical compartment more than 20% at the highest tested dose at 6 h post-exposure. Ag200 and AgNWs had no visible impact on the apical compartment at 6 h post-exposure (Fig. 4a). In the basolateral compartment, at 6 h post-exposure, independently of the AgNM tested, a significant decrease of the metabolic activity was observed at the highest concentration (Fig. 4b). Similarly to the LDH assay, a recovery of the system at 24 h post-exposure was observed, with a decrease of cellular viability in the apical compartment only at the highest dose of Ag20 (Fig. 4c). Moreover, in the basolateral compartment, an increase of the metabolic activity was observed for Ag20 and AgNWs at the intermediate and high concentrations (Fig. 4d).

### Oxidative stress response

**Fig. 5:**
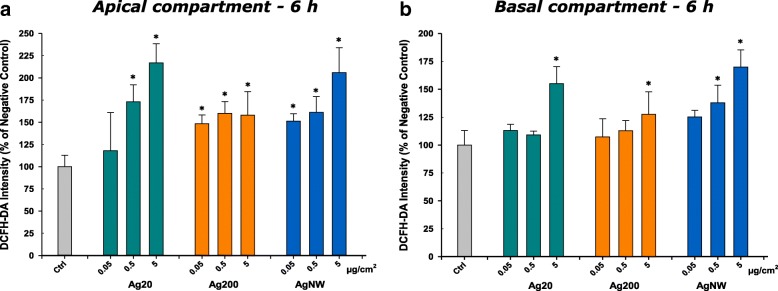
Induction of reactive oxygen species (ROS) at 6 h post-exposure. Effects of Ag20, Ag200 and AgNWs on apical (**a**) and basolateral side (**b**) after exposure to 0.05, 0.5 and 5 μg/cm^2^. The data are presented as the mean ± SD of three biological replicates. Data were expressed relative to the negative control (100%)

Reactive oxygen species (ROS) generation after the exposure to AgNMs was quantified at 6 h post-exposure. Due to its unspecific reactions with ROS, DCFH-DA assay was used to measure the overall oxidative stress [36]. All three types of AgNMs, dependent on the doses used, increased the generation of ROS in the apical and basolateral compartment (Fig. 5a and b). To further study the biological impact of AgNMs-generated ROS, the ratio between reduced (GSH) and oxidized glutathione (GSSG) was evaluated at 6 h post-exposure. None of the AgNMs tested had an impact on the GSH/GSSG ratio in the apical and basolateral side (data not shown).

### Gene expression of the pro-inflammatory and stress response elements

Based on the measurements using real-time reverse transcriptase polymerase chain reaction (RT-PCR), statistically significant changes (*P* < 0.05) were observed after exposure to AgNMs for genes encoding proteins involved in anti-oxidant response, inflammation and cellular death. Genes were further divided into two groups (stress response and inflammation) for each compartment (apical/basal) and the principal component analysis (PCA) was applied on these data subsets to highlight the differences between the tested AgNMs [37].

#### Expression of genes encoding proteins involved in the stress response in the apical compartment

**Fig. 6:**
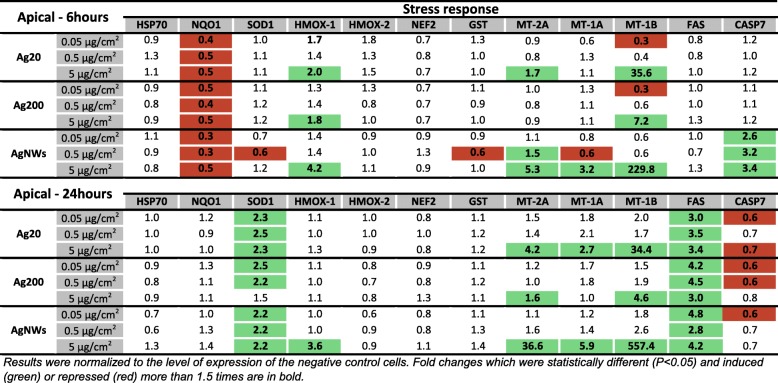
mRNA level of relevant stress markers in the apical compartment of the alveolar model at 6 h and 24 h post-exposure to Ag20, Ag200 and AgNWs

Exposure to AgNMs significantly increased the mRNA of genes encoding for *metallothioneins* (*MTs*), the most potent effect being observed for AgNWs, which increased the level of all three *MTs* at 6 h and 24 h post-exposure (Fig. 6). Moreover, exposure to AgNMs increased the mRNA of *heme-oxygenase-1* (*HMOX-1*) at the highest tested dose. At 6 h post-exposure, the effect on *HMOX-1* mRNA level increased in the same order of potency as for *MTs* (Ag200 < Ag20 < AgNWs), while at 24 h post-exposure only AgNWs at the highest dose has led to an increased level of *HMOX-1* (Fig. 6).

At 6 h post-exposure all AgNMs decreased the mRNA level of *NAD(P)H quinone dehydrogenase 1 (NQO1)*, with a return to basal level at 24 h post-exposure (Fig. 6), while at 24 h post-exposure, independently of the dose and AgNMs tested, a modest, but significant increase in the *Superoxide dismutase-1 (SOD1)* was noticed. Regarding the mRNA level of genes encoding effectors in the pro-apoptotic cascade, exposure to AgNWs increased significantly the mRNA level of *Caspase 7* (*CASP7)* at 6 h post-exposure for all concentrations tested, while at 24 h post-exposure a significant induction in the mRNA level of *FAS* was observed for all AgNMs (Fig. 6).

**Fig. 7:**
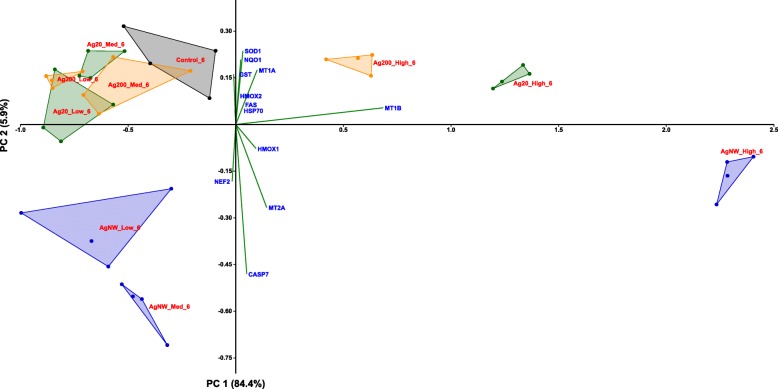
PCA analysis on the dataset of genes encoding stress response mediators at 6 h post-exposure in the apical compartment. The relative gene level (fold increase/decrease compared to negative control (gray)) of samples exposed to Ag20 (green), Ag200 (orange) and AgNWs (blue) at the three different doses (low = 0.05 μg/cm^2^, medium = 0.5 μg/cm^2^ and high = 5 μg/cm^2^) are represented on the scatter plot corresponding to PC1 and PC2

According to the PCA distribution, the effects related to the stress response were mainly dependent on the dose of AgNMs, with the highest dose treatment for each AgNMs inducing a distinctive pattern from the negative control group. Moreover, differences between the three AgNMs were observed at the highest tested dose, with AgNWs presenting the most distinctive pattern (Fig. 7; Additional file 4: Figure S3). From the differential gene expression patterns, the genes coding for *MTs* (*MT-1A, MT-1B and MT-2A*) and *HMOX-1* were the most important contributors to the existing variance in the data.

#### Expression of genes encoding proteins involved in the stress response in the basolateral compartment

**Fig. 8:**
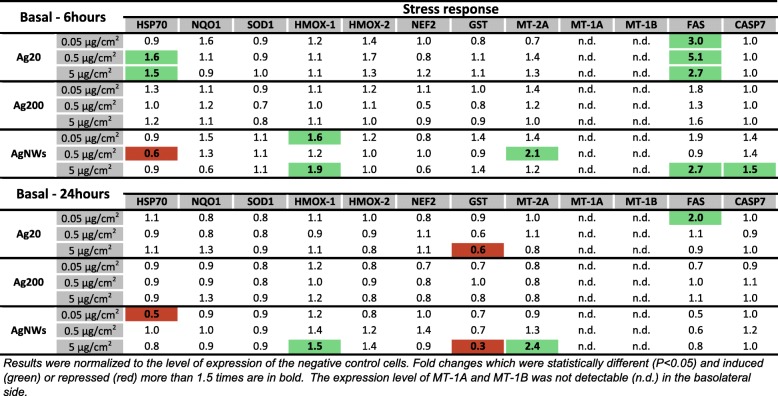
mRNA level of relevant stress markers in the basolateral compartment of the alveolar model at 6 h and 24 h post-exposure to Ag20, Ag200 and AgNWs

Exposure to AgNMs induced small changes in the mRNA level of genes encoding stress response elements in the basolateral compartment. A slight statistical increase in the mRNA of *MT-2A* was observed only for AgNWs, while in agreement with the effects observed on the apical side, AgNWs increased significantly the mRNA level of *HMOX-1* at 6 h and 24 h post-exposure (Fig. 8). At 6 h post-exposure, Ag20 transiently up-regulated the mRNA level of *Heat Shock Protein 70* (*HSP70*), a general marker for cellular stress. In addition, exposure to the highest tested dose of Ag20, and AgNWs strongly down-regulated the transcription of *Glutathione S-Transferase* (*GST*) at 24 h post-exposure (Fig. 8). Regarding the mRNA level of genes encoding pro-apoptotic proteins, exposure to Ag20 up-regulated the level of *FAS* at 6 h post-exposure at all tested doses. An up-regulation of *FAS* and *CASP7* was also observed after exposure to AgNWs at the dose of 5 μg/cm^2^ at 6 h post-exposure (Fig. 8). PCA analysis did not reveal major differences in the data subsets at both time points evaluated (Additional file 5: Figure S4 and Additional file 6: Figure S5).

#### Expression of genes encoding proteins involved in the pro-inflammatory response in the apical compartment

**Fig. 9:**
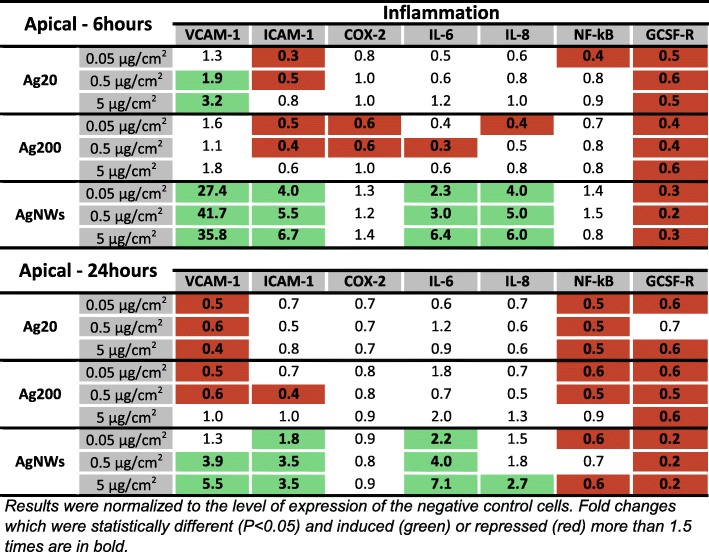
mRNA level of relevant inflammatory markers in the apical compartment of the alveolar model at 6 h and 24 h post-exposure to Ag20, Ag200 and AgNWs

Similarly to what was observed for genes encoding proteins involved in the stress response, AgNWs displayed the highest ability to increase the mRNA level of pro-inflammatory molecules in the apical compartment. At 6 h post-exposure, AgNWs increased in a dose-dependent manner the mRNA level of the pro-inflammatory *interleukins 6 and 8* (*IL-6* and *IL-8*), and of the *Intercellular Adhesion Molecule-1* (*ICAM-1)* and *Vascular Cell Adhesion Molecule-1* (*VCAM-1)* (Fig. 9). Exposure to Ag20 had also an impact on the mRNA level of *VCAM-1* at 6 h post-exposure, increasing it at the two highest concentrations tested. Opposite to this, *ICAM-1* was down-regulated by Ag20 and Ag200 at 6 h post-exposure. At 24 h post-exposure, a stable down-regulation in the mRNA level of *VCAM-1* was observed for Ag20 and Ag200 (Fig. 9).

Considering that most of the cytokines, chemokines and adhesion molecules evaluated in this study are under the regulation of the transcription factor *NF-kB*, the mRNA level of *NF-kB* was investigated. No significant effects were observed for any of the AgNMs tested at 6 h post-exposure, while at 24 h post-exposure, independently of the dose and type of AgNMs tested, a down-regulation was observed (Fig. 9).

**Fig. 10:**
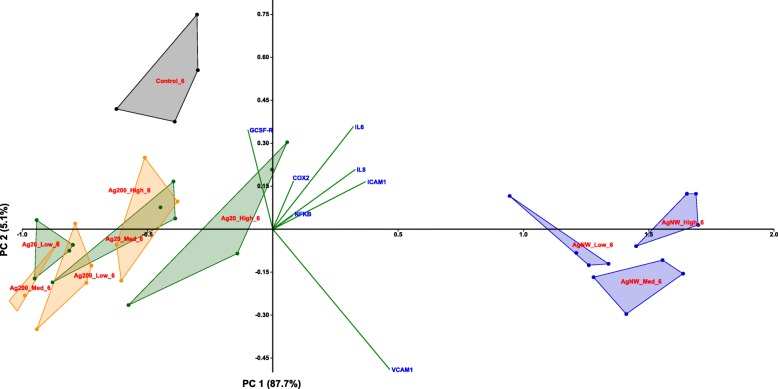
PCA analysis on the dataset of genes encoding pro-inflammatory mediators at 6 h post-exposure in the apical compartment. The relative gene level (fold increase/decrease compared to negative control (gray)) of samples exposed to Ag20 (green), Ag200 (orange) and AgNWs (blue) at the three different doses (low = 0.05 μg/cm^2^, medium = 0.5 μg/cm^2^ and high = 5 μg/cm^2^) are represented on the scatter plot corresponding to PC1 and PC2

The distinctive pro-inflammatory nature of AgNWs was further confirmed by PCA (Fig. 10; Additional file 7: Figure S6), with the most prominent differences being observed at 6 h post-exposure (Fig. 10). The most important contributing factors in the spatial distribution were the genes encoding *IL-6, IL-8, VCAM-1* and *ICAM-1.* At 24 h post-exposure, the differences between the AgNWs and the other two types of AgNMs were still observable to a lower extent, with the same genes as for 6 h post-exposure behaving as the main contributors.

#### Expression of genes encoding proteins involved in the pro-inflammatory response in the basolateral compartment

**Fig. 11:**
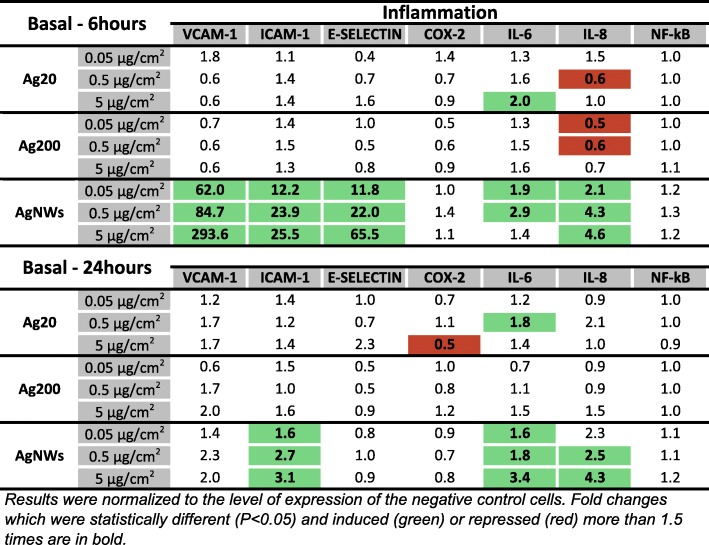
mRNA level of relevant inflammatory markers in the basolateral compartment of the alveolar model at 6 h and 24 h post-exposure to Ag20, Ag200 and AgNWs

In parallel with the responses observed in the apical compartment, exposure to AgNWs up-regulated the mRNA level of *IL-6* and *IL-8* in the basolateral compartment at both time points (Fig. 11). Conversely, a repression of *IL-8* was observed after exposure to 0.05 and 0.5 μg/cm^2^ Ag200 and 0.5 μg/cm^2^ Ag20 (Fig. 11). Regarding the mRNA level of the evaluated adhesion molecules, only AgNWs exposure induced significant alterations. Exposure to AgNWs strongly induced at 6 h post-exposure the mRNA level of all three adhesion molecules, while at 24 h post-exposure only *ICAM-1* remained significantly up-regulated (Fig. 11). The mRNA level of *NF-kB* was not modified after exposure to AgNMs (Fig. 11).

The PCA analysis confirmed the distinctive pro-inflammatory potential of AgNWs, with the genes encoding for the adhesion molecules *E-SELECTIN, VCAM-1* and *ICAM-1* being the most important contributors (Additional file 8: Figure S7 and Additional file 9: Figure S8).

### Cytokine and chemokine secretion

**Fig. 12:**
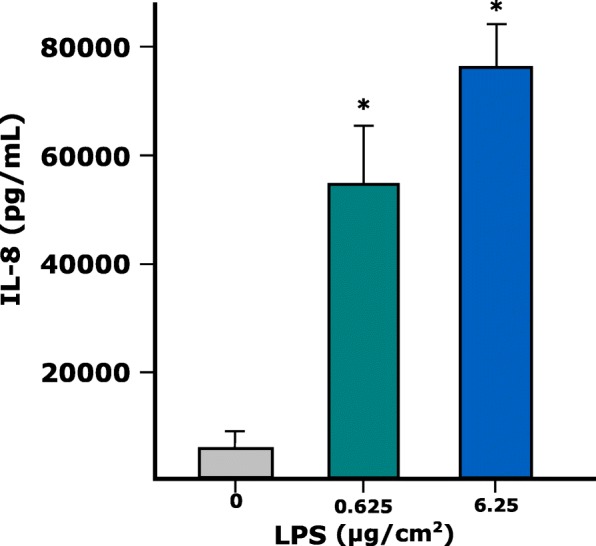
Level of IL-8 (pg/mL) in cell culture undernatants of the tetra-culture after exposure to 0.625 and 6.25 µg/cm^2 ^LPS for 24 h. Data represent the mean of three biological replicates ± SD

The ability of the model to respond to LPS exposure by secretion of pro-inflammatory cytokines was first evaluated. The ALI exposure of the alveolar model to LPS increased in a dose-dependent manner the secretion of the pro-inflammatory cytokine IL-8 (Fig. 12), without affecting the cellular viability (data not shown).

The quantification of the secreted interleukins in the cellular media from the basolateral compartment of the 3D tetra-culture alveolar model was performed after exposure to AgNWs, since only these AgNMs had an impact on the gene expression of pro-inflammatory cytokines. Exposure to AgNWs induced a dose-dependent increase in the secretion of pro−/anti-inflammatory messengers at both time points evaluated (Table 2). The lowest dose tested (0.05 μg/cm^2^) had a major impact at 6 h post-exposure, significantly increasing the expression of 6 out of 10 cytokines evaluated. Overall, after exposure to AgNWs, a more prominent biological response was observed at 6 h post-exposure, with an attenuation of the pro-inflammatory effects being observed at 24 h post-exposure for the smallest and the intermediate dose. At the highest dose tested, an increased secretion was observed for all cytokines at both time points evaluated. The absolute values expressed in pg/mL are presented in Table 2.

**Table 2:** Level of secondary messengers in cell culture undernatants of the tetra-culture after exposure to 0.05, 0.5 and 5 μg/cm^2^ AgNWs for 6 and 24 h

Cytokine	Function	6 h post-exposure				24 h post-exposure			
		Neg_control pg/mL	0.05 μg/cm^2^ pg/mL	0.5 μg/cm^2^ pg/mL	5 μg/cm^2^ pg/mL	Neg_control pg/mL	0.05 μg/cm^2^ pg/mL	0.5 μg/cm^2^ pg/mL	5 μg/cm^2^ pg/mL
IL-6	Chemotactic and pro-inflammatory cytokine; supports the growth of B cells	1.18 ± 0.10	6.22 ± 3.23	23.58 ± 1.71*	27.98 ± 5.12*	1.69 ± 0.26	1.76 ± 0.83	13.05 ± 2.46*	34.03 ± 0.80*
IL-8	Chemotactic and pro-inflammatory cytokine	738 ± 33	2216 ± 233*	2599 ± 33*	2621 ± 60*	1423 ± 74	1482 ± 447	2590 ± 22*	**2675 ± 57** *
TNF-α	Promotes the inflammation process by upregulation of other pro-inflammatory cytokines	0.83 ± 0.17	3.59 ± 1.34	8.51 ± 1.56*	11.57 ± 3.25*	1.47 ± 0.12	1.74 ± 0.42	5.44 ± 1.22*	10.70 ± 0.45*
IL-1β	Mediator of the inflammatory response	1.69 ± 0.07	3.03 ± 0.53*	5.13 ± 0.27*	5.43 ± 0.59*	3.56 ± 0.22	1.95 ± 0.59*	4.78 ± 0.71*	7.07 ± 0.41*
IL-2	Key functions in the immune system, tolerance and immunity	0.17 ± 0.10	0.62 ± 0.40*	1.65 ± 0.55*	1.53 ± 0.52*	0.34 ± 0.17	0.21 ± 0.21	1.17 ± 0.65	2.52 ± 0.46*
IL-4	Key regulator in humoral and adaptive immunity	0.006 ± 0.011	0.055 ± 0.012*	0.153 ± 0.010*	0.169 ± 0.042*	0.006 ± 0.012	0.034 ± 0.021	0.135 ± 0.022*	0.260 ± 0.024*
IL-12p70	Controls and stimulates the activities of natural killer cells	0.06 ± 0.02	0.35 ± 0.16*	0.66 ± 0.11*	0.92 ± 0.05*	0.14 ± 0.04	0.14 ± 0.11	0.75 ± 0.15*	1.40 ± 0.50*
IL-13	Key regulator in humoral and adaptive immunity proliferation	5.24 ± 0.22	11.94 ± 1.02*	17.16 ± 2.05*	17.65 ± 0.76*	8.94 ± 0.67	6.52 ± 2.08	13.55 ± 1.31*	18.36 ± 2.01*
IL-10	Anti-inflammatory cytokine; downregulates the expression of Th1 cytokines	0.11 ± 0.03	1.44 ± 1.17	2.41 ± 0.79*	2.56 ± 0.47*	0.19 ± 0.04	0.45 ± 0.12	1.63 ± 0.59*	2.57 ± 0.22*
IFN-γ	Immunostimulatory and immunomodulatory effects	0.13 ± 0.23	0.56 ± 0.59	3.12 ± 0.83*	2.53 ± 1.52*	0.28 ± 0.25	0.54 ± 0.59	2.24 ± 1.59	4.50 ± 0.90*

Data represent the mean of three biological replicates ± SD. Values statistically different from their control are in bold and marked by asterisk

### Nuclear translocation of the transcription factor NF-kB

**Fig. 13:**
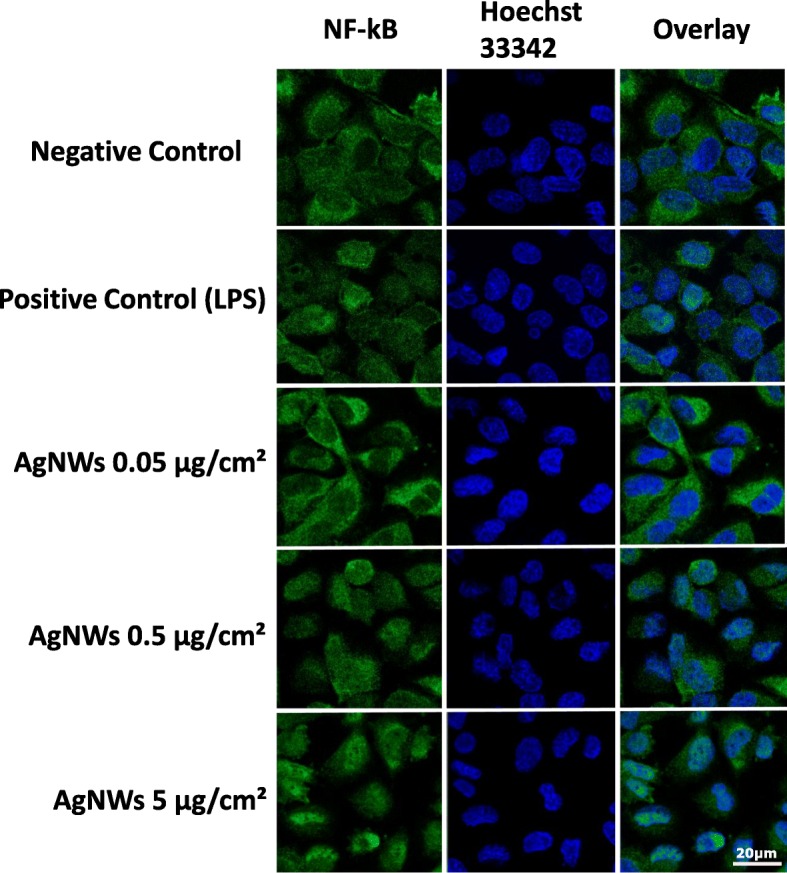
Potential of AgNWs to induce the nuclear translocation of NF-kB in endothelial cells at 4 h post-exposure. The alveolar model was exposed to AgNWs at the three concentrations studied. Cells exposed to vehicle served as Negative Control. Positive Control was exposed to Lipopolysaccharides (LPS) at a concentration of 10 μg/mL for the same period of time. Cells were fixed and stained for NF-kB (green) and nuclei (blue)

The potential of AgNMs to induce the nuclear translocation of NF-kB, one of the major molecular pathways with pro-inflammatory function was assessed as an early event at 4 h post-exposure. All three types of AgNMs clearly induced the nuclear translocation of NF-kB at the highest tested dose of 5 μg/cm^2^ in the basolateral compartment (Fig. 13; Additional file 10: Figure S9 and Additional file 11: Figure S10). In the case of AgNWs the nuclear translocation was observed also at the intermediate dose of 0.5 μg/cm^2^ (Fig. 13).

## Discussion

The high interest of industry to incorporate AgNMs in consumer products, including electronics and medical products, and the imperative need for more realistic in vitro toxicity screening of nanomaterials has prompted the current study. We aimed to investigate the distinctive biological effects elicited by two different sizes of spherical silver particles (Ag20 and Ag200) and of PVP-coated silver nanowires (AgNWs) in a 3D tetra-culture in vitro model, representative for the alveolar barrier using the Vitrocell™Cloud System. The second objective was to investigate the influence of the AgNMs shape on biological responses. Only a few scientific articles studied the biological effects of AgNWs, and to the best of our knowledge, none investigated the biological effects using an ALI system.

Stable suspensions were obtained following a published dispersion protocol [20]. The dissolution of Ag^+^ from the AgNMs due to the sonication process and the further incubation for 24 h was evaluated in order to exclude Ag^+^ as a confounding variable. The sonication procedure with or without subsequent 24 h incubation, did not impact the dissolution rate for any material, with Ag^+^ representing less than < 0.2% of total Ag. Incubation with Poractant alfa, a surrogate of human lung surfactant in premature babies, had no effect on the dissolution kinetics of AgNMs. These results are in accordance with recent studies that investigated the influence of pulmonary surfactant and of other lung lining fluid components on the dissolution of AgNWs [38] and AgNPs [39]. Even though the concentration of free Ag^+^ was low in all conditions, the contribution of free Ag^+^ to the biological effects observed cannot be fully excluded.

The in situ characterization of the nebulized AgNMs revealed changes in the agglomeration state of all materials tested. The increase in size was also reported in previous studies and was attributed to the nebulization process or to drying effects [30, 31]. The sprayed AgNWs maintained their needle-like shape after aerosolization.

The calculated deposition efficiencies after nebulisation were dependent on the concentration and the type of AgNMs sprayed. AgNWs displayed the lowest deposition efficiency, most probably due to their anisotropic shape, which may have interfered with the passage through the pores present in the membrane of the Aeroneb Pro® nebulizer. Deposition efficiencies for the AgNMs were lower than what was reported for solutions of substances [40, 41], but in agreement with deposition efficiencies reported for other nanomaterials [31] using the Vitrocell™Cloud System or a similar exposure device (ALICE) [42, 43]. The fact that the deposition of sprayed AgNMs was dependent on spraying conditions, as membrane pore size or ionic strength of the buffer used, is an important additional information.

All AgNMs decreased the cell viability of the 3D tetra-culture at 6 h post-exposure, with a recovery of the system being observed at 24 h. The results obtained from the viability assays indicated the hazard of AgNMs, and are in agreement with the current literature [44, 45]. In the majority of studies using co-cultures, a higher resilience after exposure to airborne chemicals and particles when compared to mono-cultures was observed [46–48], reiterating the need for more relevant in vitro models to study the biological effects of chemicals.

Exposure to all three types of AgNMs induced the generation of ROS, as measured by the conversion of the ROS-sensitive dye DCFH-DA. The assay was performed on both the apical and endothelial side and more pronounced effects were found on the apical side. The generation of ROS, with the subsequent initiation of a pro-inflammatory response, is currently believed to be the mechanism of toxicity for many ENMs, including AgNMs [49–51].

A total number of 20 genes encoding proteins with anti-oxidant, pro-inflammatory and pro-apoptotic function were selected in order to have a broad overview of the elicited biological responses, and to further discriminate between the AgNM specific responses. All three AgNMs increased the mRNA level of the anti-oxidant *HMOX-1* in the apical compartment at 6 h post-exposure, with the highest fold increase being observed for AgNWs. This up-regulation returned to basal levels for Ag20 and Ag200, while for AgNWs it remained at similar levels at 24 h post-exposure. Moreover, exposure to AgNWs increased the mRNA level of *HMOX-1* in the endothelial cells located on the basolateral side. HMOX-1 is an enzyme with anti-inflammatory and anti-oxidant function which catalyses the degradation of the pro-inflammatory free haeme, as well as the generation of anti-inflammatory compounds, such as bilirubin and carbon monoxide [52]. Moreover, the Nrf2/HMOX-1 axis has been shown to inhibit the NF-kB pathway and to reduce endothelial activation and dysfunction by decreasing the expression of pro-inflammatory adhesion molecules on the surface of endothelial cells [53, 54]. The induction of anti-oxidant defence mechanisms after exposure to AgNWs was supported by the increased mRNA levels of the three metallothioneins (MTs) studied. MTs are small, cysteine-rich metal-binding proteins, which act as protective stress response elements by preserving homeostasis during exposure to heavy metals or oxidative stress insults [55]. Similar results were reported using various cell lines, including the respiratory cell line BEAS-2B, suggesting the universality of this defence mechanism after exposure to AgNMs [56–58]. Regarding the level of *CASP7*, a gene encoding an effector in the caspase cascade, only exposure to AgNWs had a statistically significant effect.

All AgNMs induced the nuclear translocation of the transcription factor NF-kB in endothelial cells. For AgNWs the nuclear translocation was visible starting from the intermediate dose while for the Ag20 and Ag200 it was visible only at the highest tested dose of 5 μg/cm^2^. The nuclear translocation of NF-kB is an early event in the pro-inflammatory cascade by initiating the gene expression of multiple pro-inflammatory mediators [59]. Sustained nuclear translocation of NF-kB is an important hallmark in pathophysiological respiratory conditions such as asthma and chronic obstructive pulmonary disease [60, 61]. Even though all AgNMs induced the nuclear translocation of NF-kB at the highest tested dose, major differences in the mRNA level of genes encoding pro-inflammatory messengers were observed between AgNMs. Exposure to AgNWs induced an early increase in the mRNA level of adhesion molecules in both compartments, which returned to normal levels at 24 h post-exposure. Over-expression of adhesion molecules plays an important role in the perpetuation of chronic inflammatory processes in diseases such as atherosclerosis and asthma [62, 63]. The pro-inflammatory nature of AgNWs was further confirmed by the increased levels of the genes encoding the pro-inflammatory *IL-6* and *IL-8*. A dose- and time-dependent increase in the mRNA level of *IL-6* and *IL-8* was observed for AgNWs, while for Ag20 only the highest dose induced a significantly increased level of *IL-6* in the basolateral compartment at 6 h post-exposure. As the increased transcription or decreased degradation of pro-inflammatory genes does not necessarily translate into an actual pro-inflammatory response, the quantification of a battery of ten interleukins in cellular media from the basolateral compartment was performed (Table 2). Exposure to AgNWs induced a dose- and time-dependent increase in the secretion of all interleukins evaluated. A strong response was observed at 6 h post-exposure increasing the secretion of 6 out of 10 interleukins (IL-12p70, IL-13, IL-1β, IL-2, IL-4 and IL-8), starting from the lowest dose applied. The secretion of the other interleukins at 6 h post-exposure was statistically significant increased starting from the intermediate dose of 0.5 μg/cm^2^. At 24 h post-exposure, the biological influence of 0.05 μg/cm^2^ AgNWs was not different from the negative control, except for IL-1β, which was significantly decreased. Moreover, for the majority of interleukins evaluated, at the lowest tested dose of AgNWs, higher absolute concentration values were observed at 6 h post-exposure than at 24 h post-exposure, suggesting the existence of a transient acute response after AgNWs exposure. Conversely, at 5 μg/cm^2^ higher concentrations for each interleukin were observed at 24 h post-exposure. The ability of AgNWs to induce the secretion of pro-inflammatory mediators such as IL-8 and IL-1β was previously reported, albeit under submerged exposure and at least one order of magnitude higher doses [64, 65].

Co-cultures of epithelial cells with immune cells have been shown to have an increased relevance in the evaluation of inhalable chemicals as they better preserve the inflammatory potential observed in vivo [22, 66, 67]. The bidirectional communication between epithelial and immune cells established by the direct cell contact or by soluble mediators is considered to be the main reason behind this increased relevance in the evaluation of the pro-inflammatory potential [22, 66, 67]. In the current study, the THP-1 monocytes were differentiated into macrophage-like cells by PMA treatment for 2 days with a subsequent 4 days of rest in medium without PMA. Based on the current literature and our experience, rested macrophages can respond better to stimuli then un-rested macrophages which are over-stimulated after the treatment with the differentiation factors and can no longer respond to other stimuli [68, 69]. Moreover, the basal inflammatory state of the tetra-culture by using rested macrophages was lower than in the previously described model [22]. The secretion of IL-4, IL-10 and IL-13, three interleukins which are mainly representative for an anti-inflammatory or regulatory response, increased in the same manner as the other interleukins with a pro-inflammatory function [70]. This auto-regulatory nature of the inflammatory process is further supported by the detailed gene expression of pro-inflammatory mediators observed. The early increase in the mRNA level of the pro-inflammatory cytokines and adhesion molecules at 6 h post-exposure was followed at 24 h by a return to basal level. In normal circumstances, different subpopulations of macrophages with a pro-inflammatory (M1 subtype) or an anti-inflammatory phenotype (M2 subtype) are present at the alveolar level, and neither the M1 or M2 subtype is fully representative for the entire population of healthy macrophages in native tissue [71]. Moreover, these different macrophage phenotypes are present in the lung tissue during the initiation and resolution of the inflammatory processes [72]. The increased secretion of both anti-inflammatory and pro-inflammatory cytokines observed in the current study highlights the capacity of the described in vitro model to reflect the intricate biological responses orchestrated by native respiratory epithelia.

Taking into account the overall results obtained, shape specific effects were observed, with AgNWs displaying the highest pro-inflammatory potential, while inducing the biological anti-oxidant defence mechanisms. In vivo and in vitro studies found that long fibres are more pathogenic than short ones, with a threshold for the induction of the pro-inflammatory responses of up to 15 μm in in vivo studies, and 5 μm in in vitro studies [73–75]. High aspect ratio nanoparticles, including AgNWs have been shown to induce frustrated phagocytosis, due to the inability of macrophages to engulf and further digest long fibres, this process being associated with a release of pro-inflammatory molecules which can cause extensive damage in the surrounding tissues and cells [73–76]. Even though we cannot attribute the higher pro-inflammatory potential of AgNWs to frustrated phagocytosis as the AgNWs sprayed were less than 5 μm in length, based on the results obtained in the dissolution experiments, the pro-inflammatory nature of AgNWs is due to their specific shape and not to the extra-cellular dissolution of Ag^+^. The increased intracellular dissolution of AgNWs due to their higher internalization or physical penetration is a possible explanation for the more pronounced effects of AgNWs. The above-mentioned hypothesis is supported by a recent study describing the ability of AgNWs to enter the cells through a “needle-like” insertion mechanism [77]. Moreover, the internalized AgNWs were shown to be degraded intracellularly and to release Ag^+^ over several days after the internalization [77].

## Conclusions

The proper characterization of the physicochemical properties at the point of exposure is a critical issue when evaluating the biocompatibility of ENMs as changes during dispersion and exposure can occur. By directly exposing a complex 3D alveolar model to AgNMs on a large dynamic range (0.05–5 μg/cm^2^) significant changes related to cytotoxicity, anti-oxidant defence and pro-inflammatory responses were observed. Starting from 0.05 μg/cm^2^, a dose representative for an acute exposure in a high exposure occupational scenario, AgNWs induced significant biological changes indicative for a pro-inflammatory response. Even though we tested the acute responses of doses where the highest one is in fact corresponding to lifetime exposure, long-term exposures using achievable acute doses are still needed in order to reveal the impact of AgNMs during chronic exposure.

## Methods

### Cell culture

The 3D-tetraculture alveolar model was assembled as published [26], with minor modifications. Millicell® cell culture inserts (surface area 0.3cm^2^; 1 μm pore size; PET) were used to obtain the tetra-culture system as follows:

On day 1, human endothelial Ea.hy 926 cells were seeded on inverted inserts (3.6 × 10^4^ cells/insert). After 4 h, the inserts were returned to their normal orientation and human alveolar epithelial A549 cells were seeded (2.5 × 10^4^ cells/insert) in the apical compartment. Medium in the basolateral and apical compartment was adjusted to 900 μL and 200 μL, respectively. The cells were left for an additional 3 days to reach confluency on the insert membrane. On day 5, rested differentiated macrophages (RDM) derived from human THP-1 cell line (3.6 × 10^4^ cells/insert) and human mast cells HMC-1 (2.5 × 10^4^ cells/insert) were added in the apical compartment. After a 4 h period in which the attachment of HMC-1 and RDM cells was achieved, the medium from the apical compartment was discarded and the complete alveolar model was left at air-liquid interface (ALI) for 24 h before the exposure to silver nanomaterials (AgNMs). The RDM were previously obtained by treating THP-1 cells for 2 days with phorbol-12-myristat-13-acetate (PMA) and another 4 days of “resting” by culturing the cells in medium without PMA. The final medium used for the tetra-culture contained only 1% FBS in order to limit the proliferation of HMC-1 cells. The complete media for mono-cultures and tetra-cultures are presented in Additional file 2: Table S2.

### Particle characterization and preparation of dispersions

Two spherical silver particles with a diameter of 20 nm (Ag20) and 200 nm (Ag200), and PVP-coated AgNWs (PL-AgW50-10 mg) with an average diameter of 40–50 nm and a length of up to 50 μm were purchased as powders from PlasmaChem GmbH (Berlin, Germany). Brunauer–Emmett–Teller (BET) isotherm was used to obtain the specific surface area of the Ag20 and Ag200 in a dry state, while in the case of AgNWs, the analysis was not possible due to surface modifications as communicated by the provider. A standardized protocol was used to obtain stable suspensions for Ag20, Ag200 and AgNWs [20]. The size and zeta potential of the AgNMs suspensions (suspended in a mixture 1:1000 of PBS and double distilled water (ddH_2_O)) were measured by dynamic light scattering (Zetasizer Nano Series, Malvern Instruments Ltd., Worcestershire, UK) during three independent measurements. For the preparation of the particle dispersions, the delivered acoustic power of the probe sonicator (UP200S) in a continuous mode and an amplitude of 50% was 3.75 J/s (Additional file 12: Figure S11), while the Critical Delivered Sonication Energy (DSE_CR_) for Ag20, Ag200 and AgNWs was 185 J/mL, 375 J/mL and 375 J/mL, respectively (Additional file 13: Figure S12). The stability of the particle suspensions was not assessed over longer periods of time, as fresh suspensions were prepared before each experiment.

### Release of silver ions after sonication, and after the incubation with alveolar surfactant (Curosurf®)

Prior to exposure of the in vitro model and the evaluation of the biological responses elicited by AgNMs, the release of soluble silver due to the sonication process alone and after incubation with alveolar surfactant was evaluated. In brief, suspensions of AgNMs at 5000 μg/mL prepared in a mixture of PBS and ddH_2_O (1 to 1000) were sonicated to achieve the AgNM specific DSE_CR_. Aliquots from these suspensions were taken immediately after the sonication and at 24 h post-sonication. For total silver species, an aliquot from the well-dispersed suspension was digested in 65% HNO_3_ to obtain a homogenous silver solution which was further diluted in 1% HNO_3_ to achieve the concentrations needed for ICP-MS measurement. For soluble silver quantification, aliquots from the well-dispersed suspension were centrifuged for 40 mins at 4000 g using centrifugal filter devices with a 3 kDa cut-off (Amicon ultra-4, Millipore, Ireland). The ultra-filtrates were further diluted in 1% HNO_3_ and the soluble silver species were evaluated by ICP-MS (Elan DRC-e, Perkin Elmer, Waltham, MA, USA).

In addition to the sonication process, which was proposed to have a significant impact on the dissolution of AgNMs, the influence of alveolar surfactant on the leaching of Ag^+^ from the AgNMs was evaluated. Poractant alfa, a natural pulmonary surfactant isolated from porcine lungs, with properties similar to human pulmonary surfactant was kindly donated by Chiesi Farmaceutici (Parma, Italy). AgNMs were incubated for 24 h at 37 °C with Poractant alfa diluted 1:100 in ddH_2_O in order to simulate the interaction between the surfactant produced by the alveolar model and the sprayed AgNMs. Soluble silver species were further quantified as previously described.

### Exposures at air-liquid interface to aerosols of AgNMs

The commercial available Vitrocell™Cloud System (Vitrocell™, Waldkirch, Germany) was used to mimic the inhalation exposure to AgNMs. The system is equipped with an Aeroneb Pro® vibrating membrane nebulizer, which generates a dense cloud of droplets with a median aerodynamic diameter of 4.0–6.0 μm that are deposited at the bottom of the exposure chamber. Cells exposed to the vehicle (PBS and ddH_2_O 1:1000) served as negative controls.

### Characterization of the deposited AgNMs

A total volume of 200 μL of Ag20, Ag200 and AgNWs suspensions at concentrations of 50, 500 and 5000 μg/mL were sprayed to measure the deposition in terms of efficiency and uniformity. The above-mentioned concentrations were selected based on a 50% deposition yield reported for a similar exposure device (ALICE system), in order to obtain a theoretical deposited dose of 0.05, 0.5 and 5 μg/cm^2^. The deposition efficiencies and the uniformity of depositions were assessed during three independent exposures for each AgNM tested. In each exposure run, 5 surrogate inserts filled with 1 mL of ddH_2_O were exposed to the AgNM aerosols, and the total content of the inserts was retrieved in metal-free tubes for further analysis. The quantification of the deposited mass per cm^2^ was further done by measuring total silver species with ICP-MS.

The uniformity of the deposition was further evaluated by Helium Ion Microscopy (HIM), Secondary Ion Mass spectrometry (SIMS) and Enhanced Darkfield Microscopy (CytoViva®). HIM consists in scanning the sample with a finely focussed He^+^ ion beam of sub-nm resolution and detecting secondary electrons to generate images. In contrast to conventional Scanning Electron Microscopy (SEM) using an electron beam to scan the sample, HIM provides better contrast details due to smaller beam-sample interaction volumes, better depth of field and the possibility to image insulating samples without prior deposition of a conductive film. In addition, the HIM used for this study was equipped with a SIMS system developed by LIST. The integrated HIM-SIMS instrument hence allows acquiring both high-resolution structural information and elemental/chemical information from the same field of view [33, 34]. The secondary electron images were obtained with a helium beam at a beam energy of 20 keV and a beam intensity of 0.5 pA. The SIMS images were acquired with a neon beam at beam energy of 20 keV and beam intensity of 2 pA.

Suspensions of AgNMs were sprayed using the Vitrocell™Cloud System on glass coverslips for the high-resolution dark field microscopy, while for HIM-SIMS analysis silicon wafers were used.

### Cytotoxicity and metabolic impairment

Alamar Blue (AB) and Lactate Dehydrogenase Assays (LDH) were used to assess a possible cytotoxic effect at 6 h and 24 h post-exposure. For AB assay, AgNMs exposed inserts were washed with PBS and further incubated with 250 μL of medium containing 400 μM resazurin for 1 h at 37 °C and 5% CO_2_. The assay was performed on both apical and basolateral sides. Aliquots of 100 μL were taken from both compartments and transferred into a 96 well plate to measure the fluorescence intensity. The values were expressed as relative values compared to negative control.

For the LDH assays, a commercially available kit from Promega® was used according to the supplier’s manual. Briefly, after AgNMs exposure, 100 μL of medium from the basolateral compartment was incubated with 100 μL LDH-substrate mix for 10 mins with the subsequent addition of 50 μL of stop solution. For the positive control, cells were previously lysed using Triton X-100 and maximum LDH release was quantified in a similar manner. Values were expressed as a percentage of the maximum LDH release. Fluorescence readings for both assays were done using a multi-mode microplate reader in emission mode (ex: 560 nm, em: 590 nm; Tecan, Austria).

### Generation of reactive oxygen species

The oxidative stress induced by the Ag20, Ag200 and AgNWs was determined at 6 h post-exposure using the ROS sensitive dye 2′,7′-Dichlorofluorescin diacetate (DCFH-DA). Briefly, at 6 h post-exposure, cells were loaded for 30 min with 300 μL of 40 μM DCFH-DA diluted in PBS in both compartments. The DCFH-DA excess was washed with PBS and cells were lysed in both compartments with 1% sodium dodecyl sulfate (Biorad). 100 μL from each lysate were pipetted in a 96 black well plate and the fluorescent signal was measured with a multiplate reader (ex: 485 nm, em: 535 nm; Tecan, Austria).

### The biological impact of ROS on reduced and oxidized glutathione ratio

The ratio between reduced glutathione (GSH) and oxidized glutathione (GSSG) was measured using the commercially available kit GSH/GSSG-Glo® Assay from Promega, according to the manufacturer’s instruction. Briefly, at 6 h post-exposure, cell inserts were washed gently with PBS and 50 μL of Total Glutathione Lysis Reagent or Oxidized Glutathione Lysis Reagent were added on both sides of the inserts, followed by a 5 min centrifugation at 600 g. 20 μL of supernatant was transferred to a white 96 well microplate and incubated for 30 mins with 50 μL of Luciferin Generation Reagent at room temperature (RT) in the dark. After the incubation time, 100 μL of Luciferin Detection reagent was added and the luminescence was read after 15 min using a multi-mode microplate reader.

### Real-time reverse transcriptase polymerase chain reaction (real-time RT-PCR) of pro-inflammatory and stress response markers

At 6 h and 24 h post-exposures, cells were washed twice with PBS and total RNA was isolated from the apical and basolateral compartments with RNeasy Mini Kit (Qiagen, Leusden, Netherlands) following manufacturer’s guidelines. Nanodrop ND1000 spectrophotometer (Thermo Scientific, Villebon-sur-Yvette, France) was used to assess the concentration and purity of the extracted RNA. Integrity of the extracted RNA was controlled using the Agilent 2100 Bioanalyzer (Agilent Technologies, Diegem, Belgium), with all samples displaying RIN (RNA integrity number) values above 8. cDNA were prepared from 1 μg of RNA using the following reagents: Protoscript II reverse transcriptase, murine RNase inhibitor (New England Biolabs, Ipswich, MA, US), dNTPs (Promega) and random primers (Invitrogen, Carlsbad, NM, USA) according to manufacturer’s protocol. qRT-PCR was performed on a ViiA 7 Real-Time PCR System (Thermo-Fisher, Waltham, MA, USA) using the Takyon low ROX SYBR MasterMix dTTP Blue Kit (Eurogentec, Liege, Belgium). No-Template and genomic DNA controls were added in each plate to exclude possible contamination from the used reagents, and with genomic DNA. Three technical replicates were performed for each sample. Four reference genes *B2M, HPRT1, YWHAZ* and *SDHA* were validated as stable candidates between experimental samples using GeNorm in the Biogazelle qBase PLUS software. The relative gene expression was calculated based on the ^ΔΔ^CT method using the Biogazelle qbase PLUS software 2.5. The list of primers (Eurogentec Liege, Belgium) for the reference and interest genes is summarized in Additional file 2: Table S3. A biological significant impact on gene expression was considered when values were statistically different (*P* < 0.05, Mann-Whitney) and when the fold change relative to the negative control was below or above the arbitrary threshold of 1.5.

### Cytokine and chemokine secretion

The ability of the model to increase the secretion of pro-inflammatory cytokines after exposure to lipopolysaccharide (LPS) was evaluated. The alveolar model was exposed directly at the ALI to 0.625 and 6.25 μg/cm^2^ LPS and the level of IL-8, a known pro-inflammatory cytokine, was measured from the cellular undernatants at 24 h post-exposure. The measurement was done using a Quantikine ELISA assay kit (R&D systems, Minneapolis MN, USA), according to the manufacturer’s instructions. The absorbance was measured using a Synergy 2 Multi-Mode Microplate Reader (BioTek).

At 6 h and 24 h post-exposure to AgNWs, aliquots of media were taken from the basolateral compartment and stored at − 80 °C until analysis. The concentrations of IL-1β, IL-2, IL-4, IL-6, IL-8, IL-10, IL-12p70, IL-13, IFN-γ and TNF-α were measured using a V-PLEX pro-inflammatory Panel 1 Human kit according to the manufacturer’s instructions (Meso Scale Diagnostics, Rockville, US). Quantification of the inflammatory cytokines was performed on a MSD QuickPlex SQ 120 (Meso Scale Diagnostics) following the manufacturer’s protocols.

### Confocal microscopy

At 4 h post-exposure, the inserts were washed twice with PBS, and fixed for 10 min using 4% buffered paraformaldehyde at RT. After fixation, cells were washed and incubated with 10% bovine serum albumin (BSA) in PBS (*w*/*v*).

For the nuclear translocation of NF-kB, the endothelial cells from the basolateral compartment were incubated for 60 min, in the dark, at RT with the primary antibody for NF-kB (ab16502; Abcam, UK) diluted 1:1000 in 1% BSA in PBS (w/v). A secondary antibody conjugated with the fluorophore DyLight633 (AS09 633; Agrisera, Vännas, Sweden) was used. Tetra-cultures exposed for the same period of time to 10 μg/mL Lipopolysaccharides (LPS) were used as positive control.

For a comparison before and after exposure to AgNWs, a staining similar to the one described previously for NF-kB translocation was used on the apical part of the alveolar model. The macrophage population was stained using the primary antibody for CD11b (AM32402PU-N - ACRIS) diluted 1:10 and a secondary antibody conjugated with the fluorophore AlexaFluor488. The cellular membranes were counterstained with cell mask deep red (C10046; Invitrogen, Leusden, the Netherlands) and nuclei with Hoechst 33342.

A Zeiss LSM 880 with airyscan with an inverted Zeiss microscope (Axiovert 200 M, Lasers: HeNe 633 nm, HeNe 543 nm, Ar 488 nm and Diode 405 nm; Zeiss, Jena, Germany) was used for image acquisition. Image processing and visualization was performed using the Zeiss Software ZEN 2011.

### Statistics

All data are presented as the mean ± standard deviation (SD) of at least three biological replicates unless otherwise specified. Statistical analysis was done using SigmaPlot v13.0 software. A one-way analysis of variance (ANOVA) with a subsequent Dunnett’s post-hoc test was performed. Differences between values were considered statistically significant when *P* < 0.05(*). For principal component analysis (PCA) the free software Past3 (https://folk.uio.no/ohammer/past/) was used.

## Additional files

Additional file 1: **Figure S1.** Secondary electron image of pristine AgNWs (bar scale 500 nm) obtained on the Helium Ion Microscopy – Secondary Ion Mass Spectrometry (HIM-SIMS) instrument. Samples were deposited on a Silicon wafer. (PDF 135 kb)

Additional file 2: **Table S1.** Intra- and inter-exposure variability calculated based on the deposited quantities of Ag20, Ag200 and AgNWs during three independent exposures (Exp_1, Exp_2 and Exp_3), using the Vitrocell™Cloud System.**Table S2.** Complete media for mono-cultures and tetra-culture. **Table S3.** List of primer sequences used for qRT-PCR experiments on the apical and basolateral side of the alveolar model. (DOCX 23 kb)

Additional file 3: **Figure S2.** The alveolar model was exposed to AgNWs at the highest tested concentration (5 μg/cm^2^). Cells exposed to vehicle served as Negative Control. Cells were fixed and stained for cellular membranes (red) and nuclei (blue). Macrophage population was stained with CD11b (green). (PDF 3939 kb)

Additional file 4: **Figure S3.** PCA analysis on the dataset of genes encoding stress response mediators at 24 h post-exposure in the apical compartment. The relative gene level (fold increase/decrease compared to negative control (gray)) of samples exposed to Ag20 (green), Ag200 (orange) and AgNWs (blue) at the three different doses (low = 0.05 μg/cm^2^, medium = 0.5 μg/cm^2^ and high = 5 μg/cm^2^) are represented on the scatter plot corresponding to PC1 and PC2. (PDF 30 kb)

Additional file 5: **Figure S4.** PCA analysis on the dataset of genes encoding stress response mediators at 6 h post-exposure in the basal compartment. The relative gene level (fold increase/decrease compared to negative control (gray)) of samples exposed to Ag20 (green), Ag200 (orange) and AgNWs (blue) at the three different doses (low = 0.05 μg/cm^2^, medium = 0.5 μg/cm^2^ and high = 5 μg/cm^2^) are represented on the scatter plot corresponding to PC1 and PC2. (PDF 29 kb)

Additional file 6: **Figure S5.** PCA analysis on the dataset of genes encoding stress response mediators at 24 h post-exposure in the basal compartment. The relative gene level (fold increase/decrease compared to negative control (gray)) of samples exposed to Ag20 (green), Ag200 (orange) and AgNWs (blue) at the three different doses (low = 0.05 μg/cm^2^, medium = 0.5 μg/cm^2^ and high = 5 μg/cm^2^) are represented on the scatter plot corresponding to PC1 and PC2. (PDF 21 kb)

Additional file 7: **Figure S6.** PCA analysis on the dataset of genes encoding pro-inflammatory mediators at 24 h post-exposure in the apical compartment. The relative gene level (fold increase/decrease compared to negative control (gray)) of samples exposed to Ag20 (green), Ag200 (orange) and AgNWs (blue) at the three different doses (low = 0.05 μg/cm^2^, medium = 0.5 μg/cm^2^ and high = 5 μg/cm^2^) are represented on the scatter plot corresponding to PC1 and PC2. (PDF 22 kb)

Additional file 8: **Figure S7.** PCA analysis on the dataset of genes encoding pro-inflammatory mediators at 6 h post-exposure in the basal compartment. The relative gene level (fold increase/decrease compared to negative control (gray)) of samples exposed to Ag20 (green), Ag200 (orange) and AgNWs (blue) at the three different doses (low = 0.05 μg/cm^2^, medium = 0.5 μg/cm^2^ and high = 5 μg/cm^2^) are represented on the scatter plot corresponding to PC1 and PC2. (PDF 21 kb)

Additional file 9: **Figure S8.** PCA analysis on the dataset of genes encoding pro-inflammatory mediators at 24 h post-exposure in the basal compartment. The relative gene level (fold increase/decrease compared to negative control (gray)) of samples exposed to Ag20 (green), Ag200 (orange) and AgNWs (blue) at the three different doses (low = 0.05 μg/cm^2^, medium = 0.5 μg/cm^2^ and high = 5 μg/cm^2^) are represented on the scatter plot corresponding to PC1 and PC2. (PDF 21 kb)

Additional file 10: **Figure S9.** Potential of Ag20 to induce the nuclear translocation of NF-kB in endothelial cells at 4 h post-exposure. The alveolar model was exposed to Ag20 at the three concentrations studied. Cells exposed to vehicle served as Negative Control. Positive Control was exposed to Lipopolysaccharides (LPS) at a concentration of 10 μg/mL for the same period of time. Cells were fixed and stained for NF-kB (green) and nuclei (blue). (PDF 1722 kb)

Additional file 11: **Figure S10.** Potential of Ag200 to induce the nuclear translocation of NF-kB in endothelial cells at 4 h post-exposure. The alveolar model was exposed to Ag200 at the three concentrations studied. Cells exposed to vehicle served as Negative Control. Positive Control was exposed to Lipopolysaccharides (LPS) at a concentration of 10 μg/mL for the same period of time. Cells were fixed and stained for NF-kB (green) and nuclei (blue). (PDF 1773 kb)

Additional file 12: **Figure S11.** Calorimetric calibration of the UP200S sonicator. 50 mL of ddH_2_O were sonicated at amplitude of 50% in a continuous mode up to 4800 s and the difference in temperature was measured. The plot representing the difference in temperature (ΔT) as a function of the sonication period (Δt) was constructed, and from the first linear portion, the delivered acoustic power (W = Joule/Second) was calculated. (PDF 26 kb)

Additional file 13: **Figure S12.** Determination of Critical Delivered Sonication Energy (DSE_CR_) for Ag20, Ag200 and AgNWs. The plot is presenting the mean hydrodynamic diameter as a function of the total delivered sonication energy (DSE). Hydrodynamic diameter was measured by DLS. (PDF 19 kb)
