# Supplementary figures and images for: In vitro exposure of a 3D-tetraculture representative for the alveolar barrier at the air-liquid interface to silver particles and nanowires
Source: Part Fibre Toxicol. 2019 Apr 2;16:14. doi: 10.1186/s12989-019-0297-1 (PMC6444883; doi:10.1186/s12989-019-0297-1)

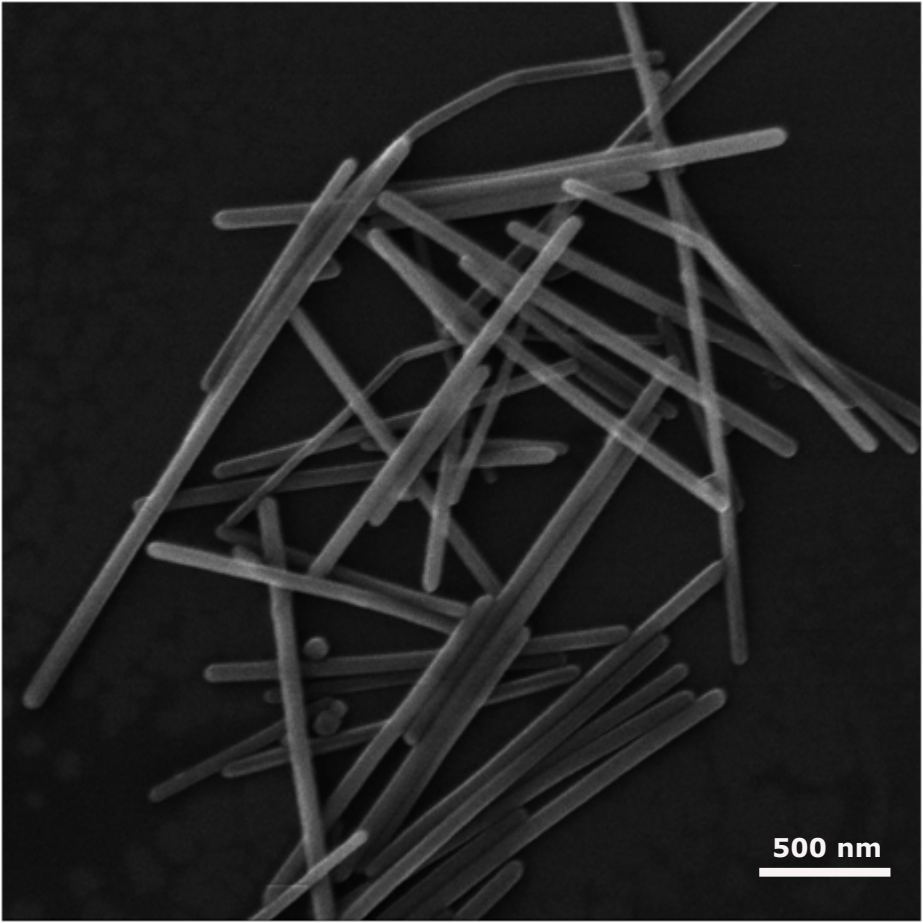

Supplement: Supplementary file 1 — Figure S1. Secondary electron image of pristine AgNWs (bar scale 500 nm) obtained on the Helium Ion Microscopy – Secondary Ion Mass Spectrometry (HIM-SIMS) instrument. Samples were deposited on a Silicon wafer. (PDF 135 kb) [file 12989_2019_297_MOESM1_ESM.pdf]

Negative Control

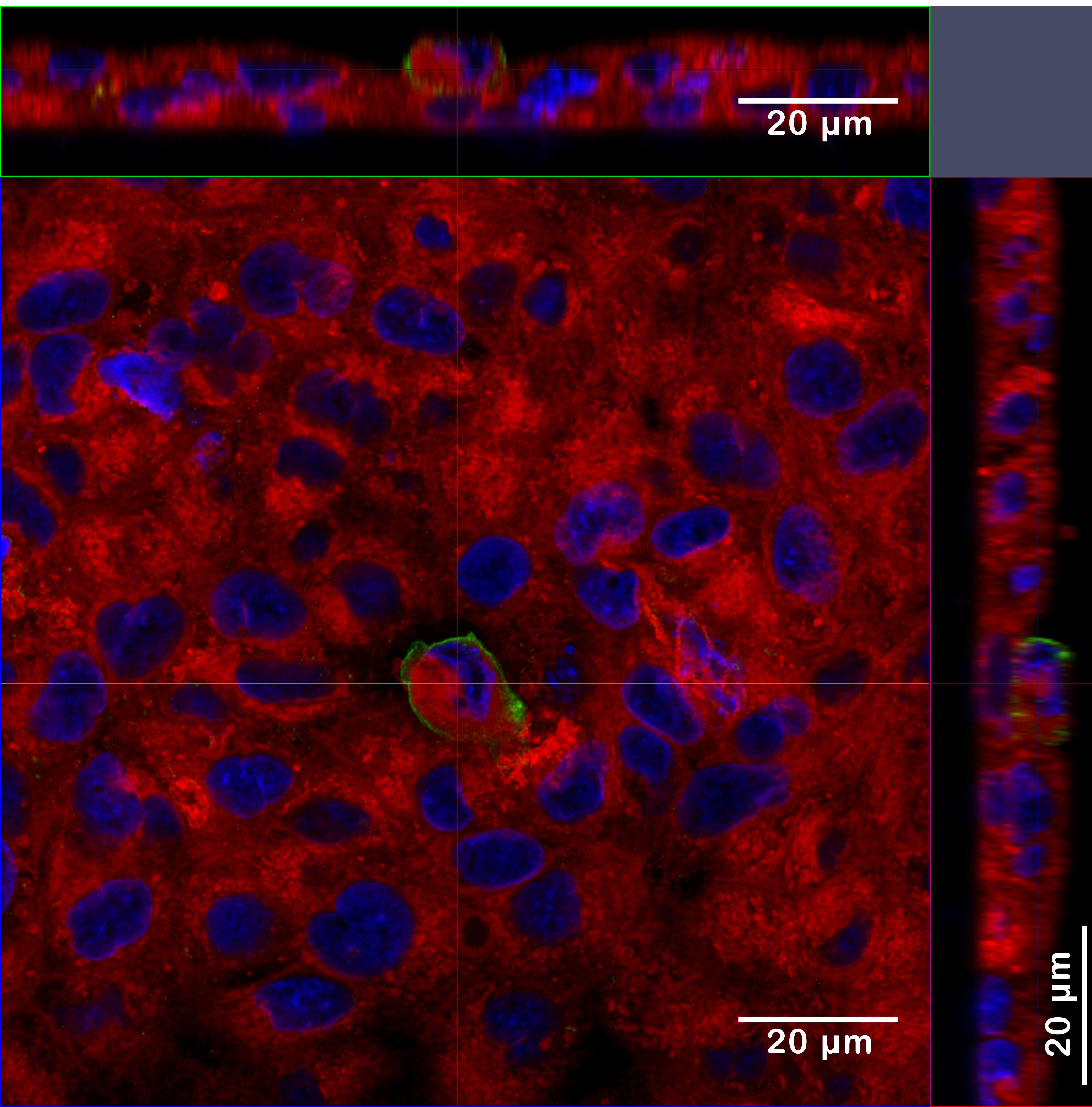

AgNWs 5  $\mu\text{g}/\text{cm}^2$

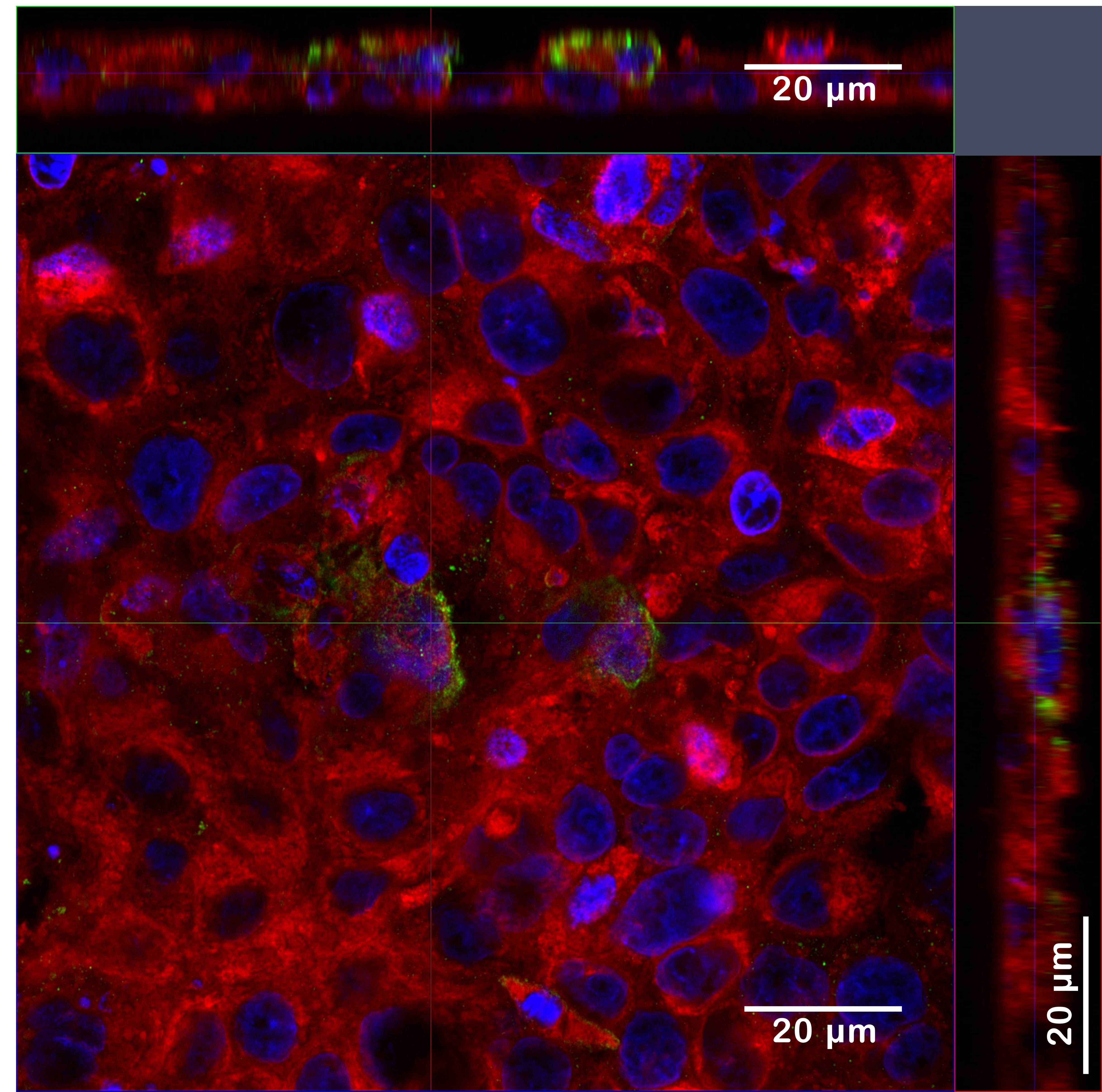

Supplement: Supplementary file 3 — Figure S2. The alveolar model was exposed to AgNWs at the highest tested concentration (5 μg/cm2). Cells exposed to vehicle served as Negative Control. Cells were fixed and stained for cellular membranes (red) and nuclei (blue). Macrophage population was stained with CD11b (green). (PDF 3939 kb) [file 12989_2019_297_MOESM3_ESM.pdf]

PC 2 (6.1%)

PC 1 (82.3%)

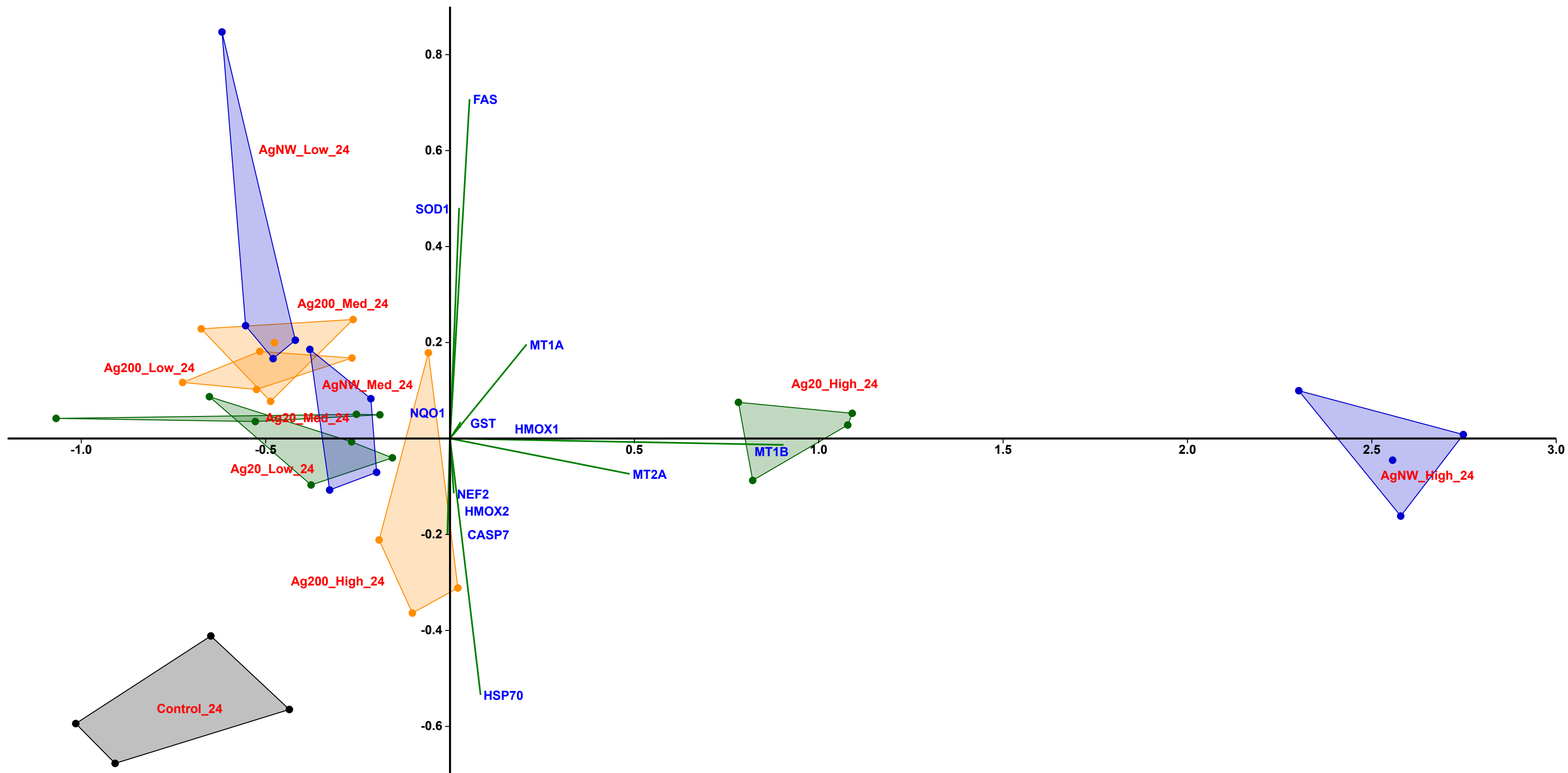

Supplement: Supplementary file 4 — Figure S3. PCA analysis on the dataset of genes encoding stress response mediators at 24 h post-exposure in the apical compartment. The relative gene level (fold increase/decrease compared to negative control (gray)) of samples exposed to Ag20 (green), Ag200 (orange) and AgNWs (blue) at the three different doses (low = 0.05 μg/cm2, medium = 0.5 μg/cm2 and high = 5 μg/cm2) are represented on the scatter plot corresponding to PC1 and PC2. (PDF 30 kb) [file 12989_2019_297_MOESM4_ESM.pdf]

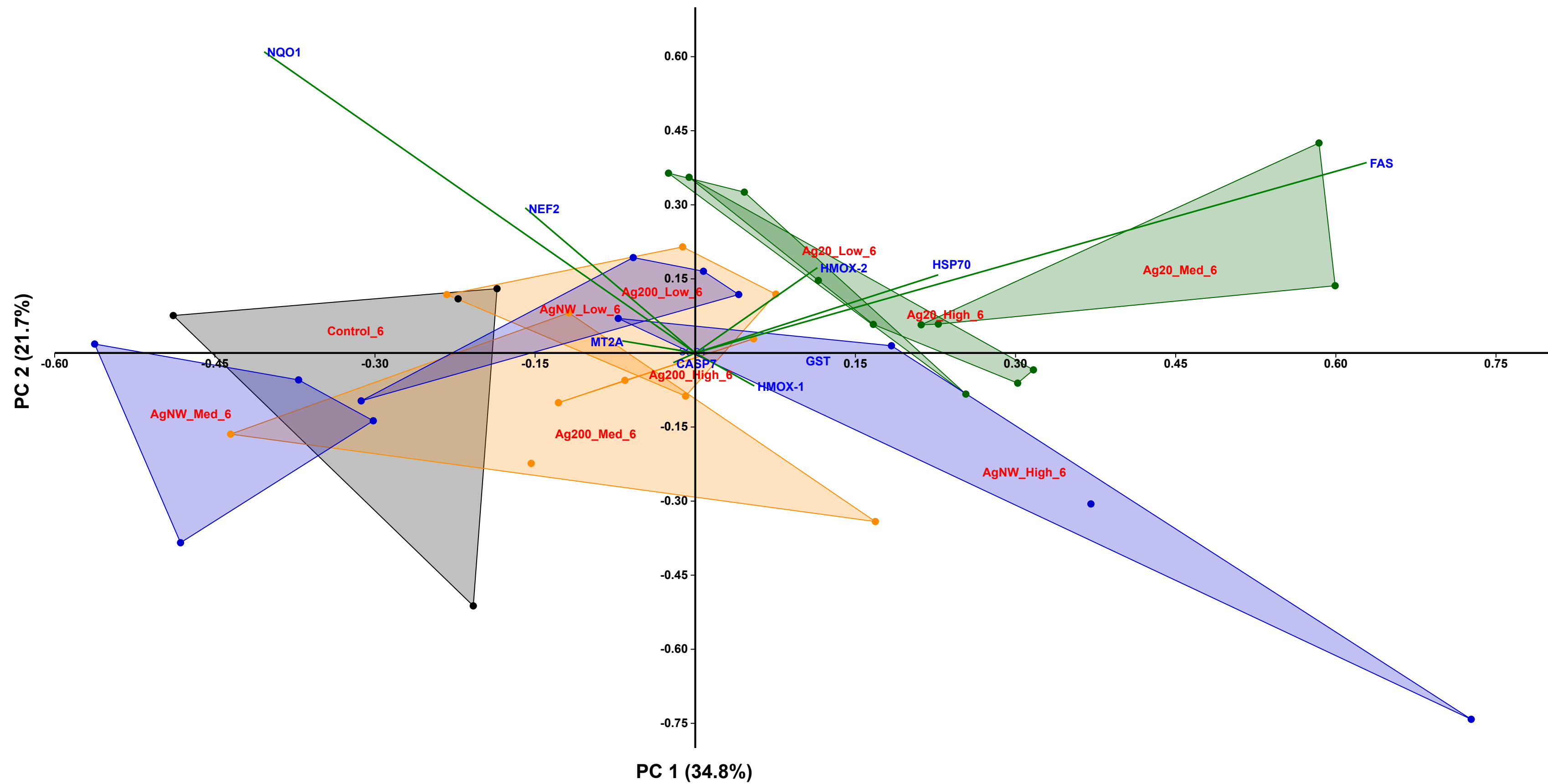

Supplement: Supplementary file 5 — Figure S4. PCA analysis on the dataset of genes encoding stress response mediators at 6 h post-exposure in the basal compartment. The relative gene level (fold increase/decrease compared to negative control (gray)) of samples exposed to Ag20 (green), Ag200 (orange) and AgNWs (blue) at the three different doses (low = 0.05 μg/cm2, medium = 0.5 μg/cm2 and high = 5 μg/cm2) are represented on the scatter plot corresponding to PC1 and PC2. (PDF 29 kb) [file 12989_2019_297_MOESM5_ESM.pdf]

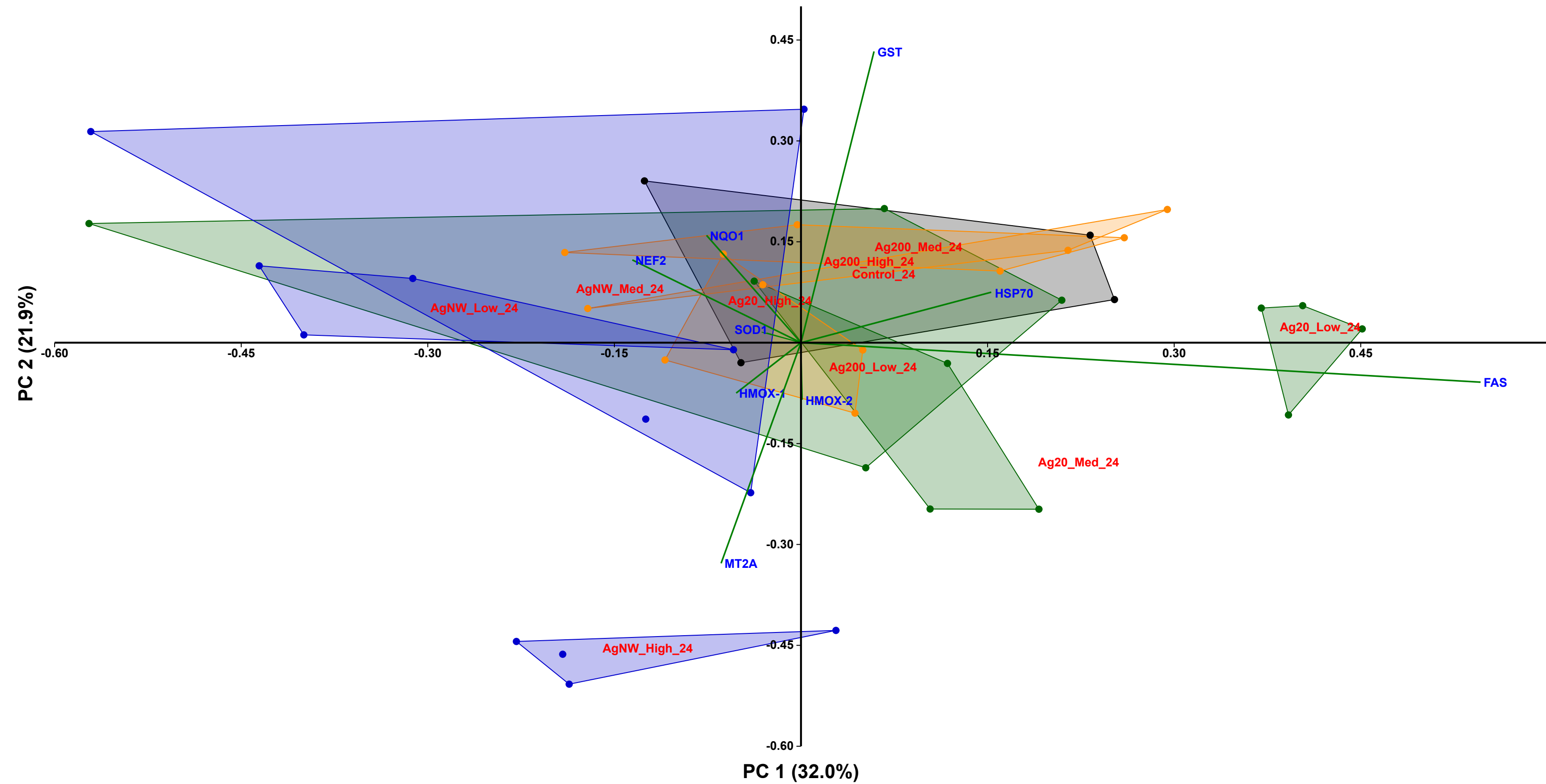

Supplement: Supplementary file 6 — Figure S5. PCA analysis on the dataset of genes encoding stress response mediators at 24 h post-exposure in the basal compartment. The relative gene level (fold increase/decrease compared to negative control (gray)) of samples exposed to Ag20 (green), Ag200 (orange) and AgNWs (blue) at the three different doses (low = 0.05 μg/cm2, medium = 0.5 μg/cm2 and high = 5 μg/cm2) are represented on the scatter plot corresponding to PC1 and PC2. (PDF 21 kb) [file 12989_2019_297_MOESM6_ESM.pdf]

PC 2 (12.1%)

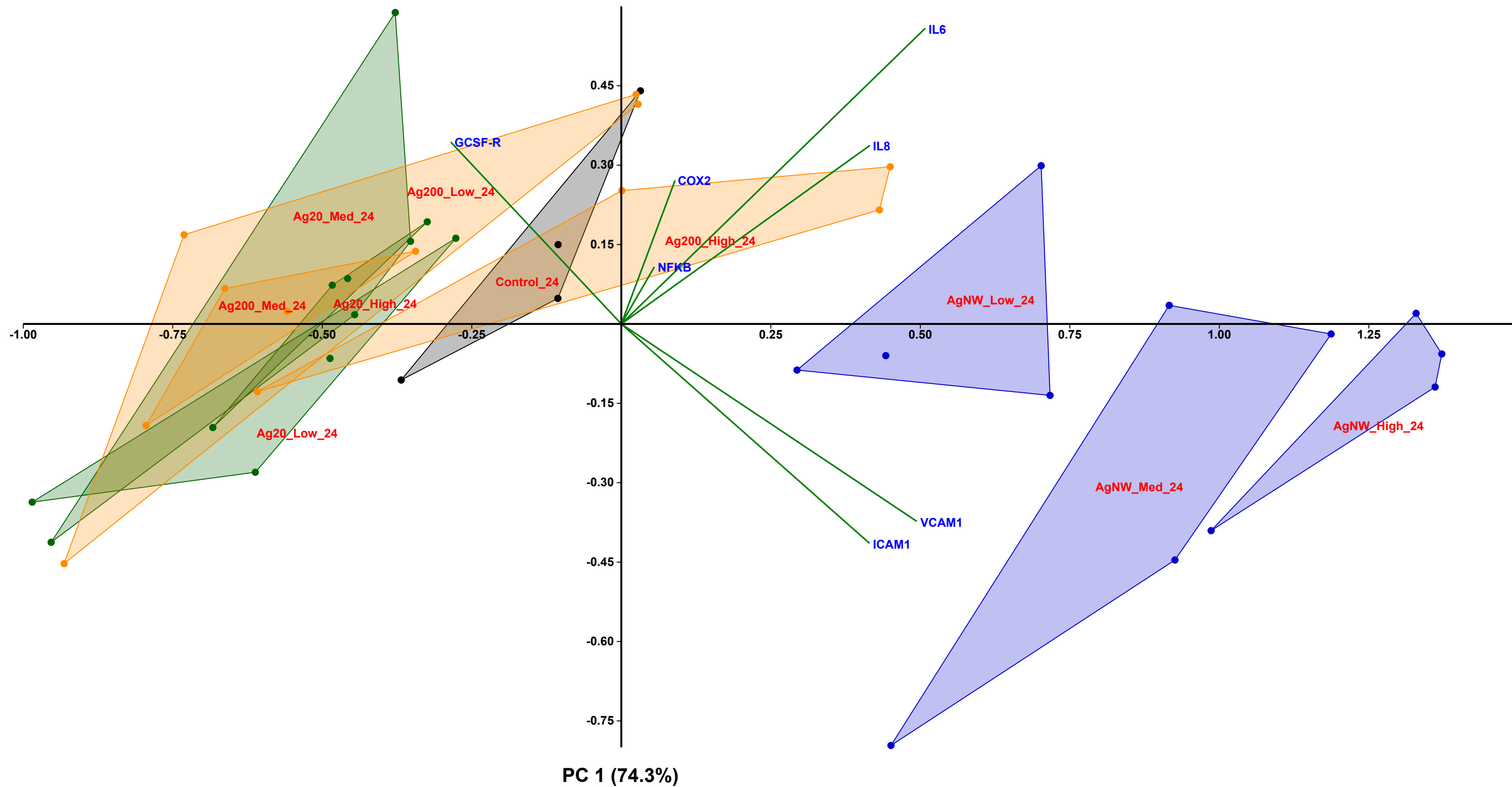

Supplement: Supplementary file 7 — Figure S6. PCA analysis on the dataset of genes encoding pro-inflammatory mediators at 24 h post-exposure in the apical compartment. The relative gene level (fold increase/decrease compared to negative control (gray)) of samples exposed to Ag20 (green), Ag200 (orange) and AgNWs (blue) at the three different doses (low = 0.05 μg/cm2, medium = 0.5 μg/cm2 and high = 5 μg/cm2) are represented on the scatter plot corresponding to PC1 and PC2. (PDF 22 kb) [file 12989_2019_297_MOESM7_ESM.pdf]

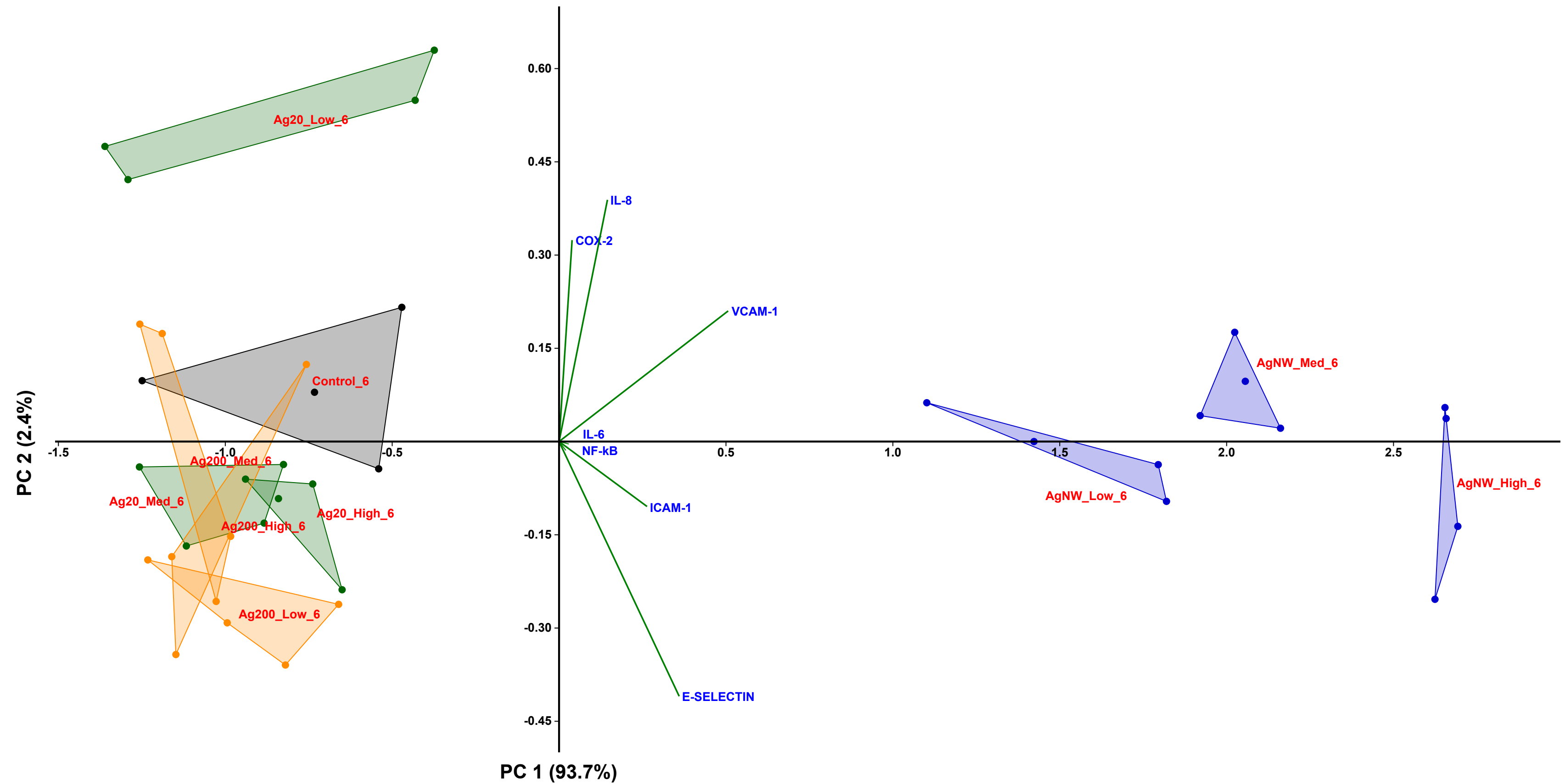

Supplement: Supplementary file 8 — Figure S7. PCA analysis on the dataset of genes encoding pro-inflammatory mediators at 6 h post-exposure in the basal compartment. The relative gene level (fold increase/decrease compared to negative control (gray)) of samples exposed to Ag20 (green), Ag200 (orange) and AgNWs (blue) at the three different doses (low = 0.05 μg/cm2, medium = 0.5 μg/cm2 and high = 5 μg/cm2) are represented on the scatter plot corresponding to PC1 and PC2. (PDF 21 kb) [file 12989_2019_297_MOESM8_ESM.pdf]

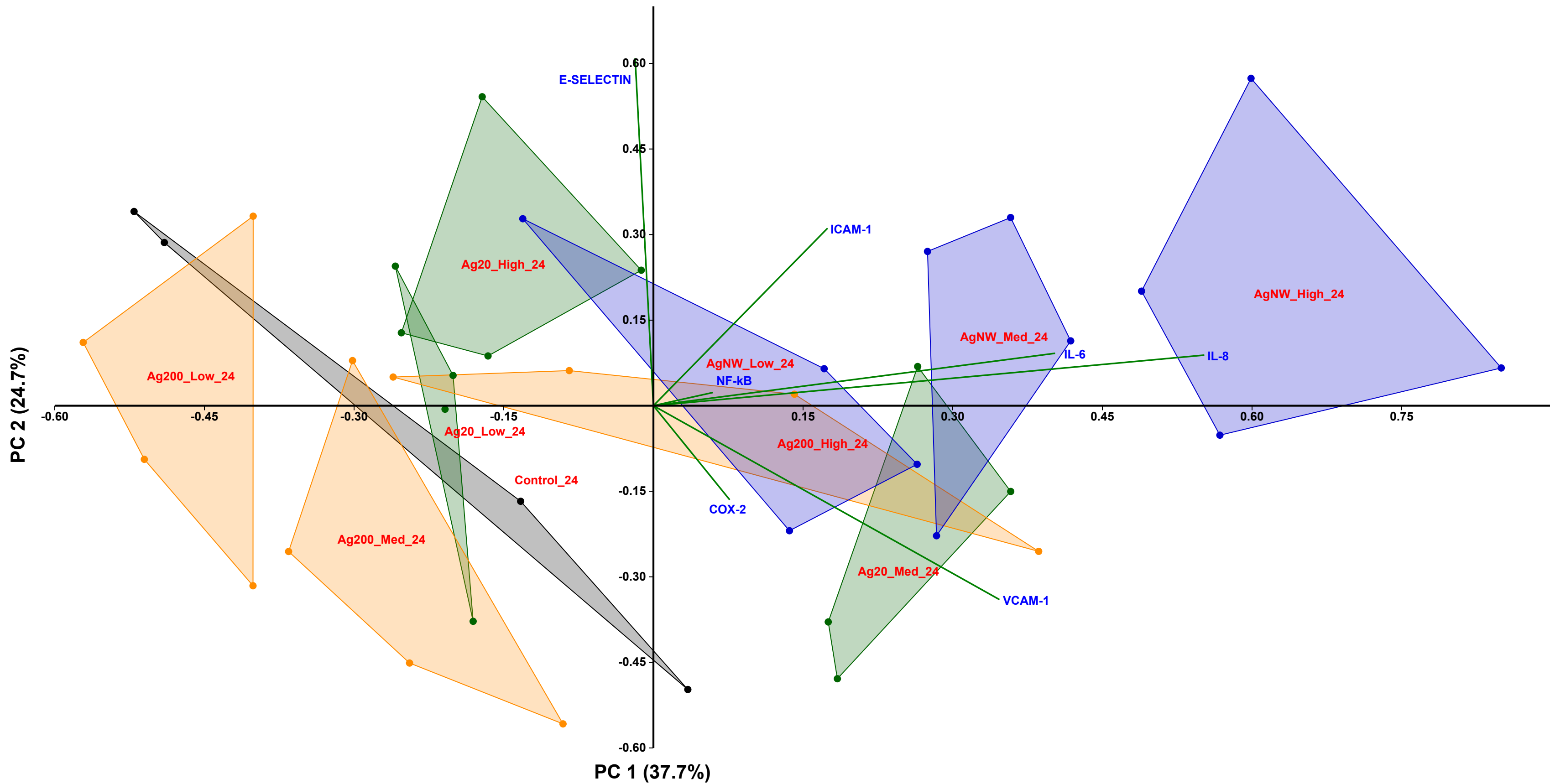

Supplement: Supplementary file 9 — Figure S8. PCA analysis on the dataset of genes encoding pro-inflammatory mediators at 24 h post-exposure in the basal compartment. The relative gene level (fold increase/decrease compared to negative control (gray)) of samples exposed to Ag20 (green), Ag200 (orange) and AgNWs (blue) at the three different doses (low = 0.05 μg/cm2, medium = 0.5 μg/cm2 and high = 5 μg/cm2) are represented on the scatter plot corresponding to PC1 and PC2. (PDF 21 kb) [file 12989_2019_297_MOESM9_ESM.pdf]

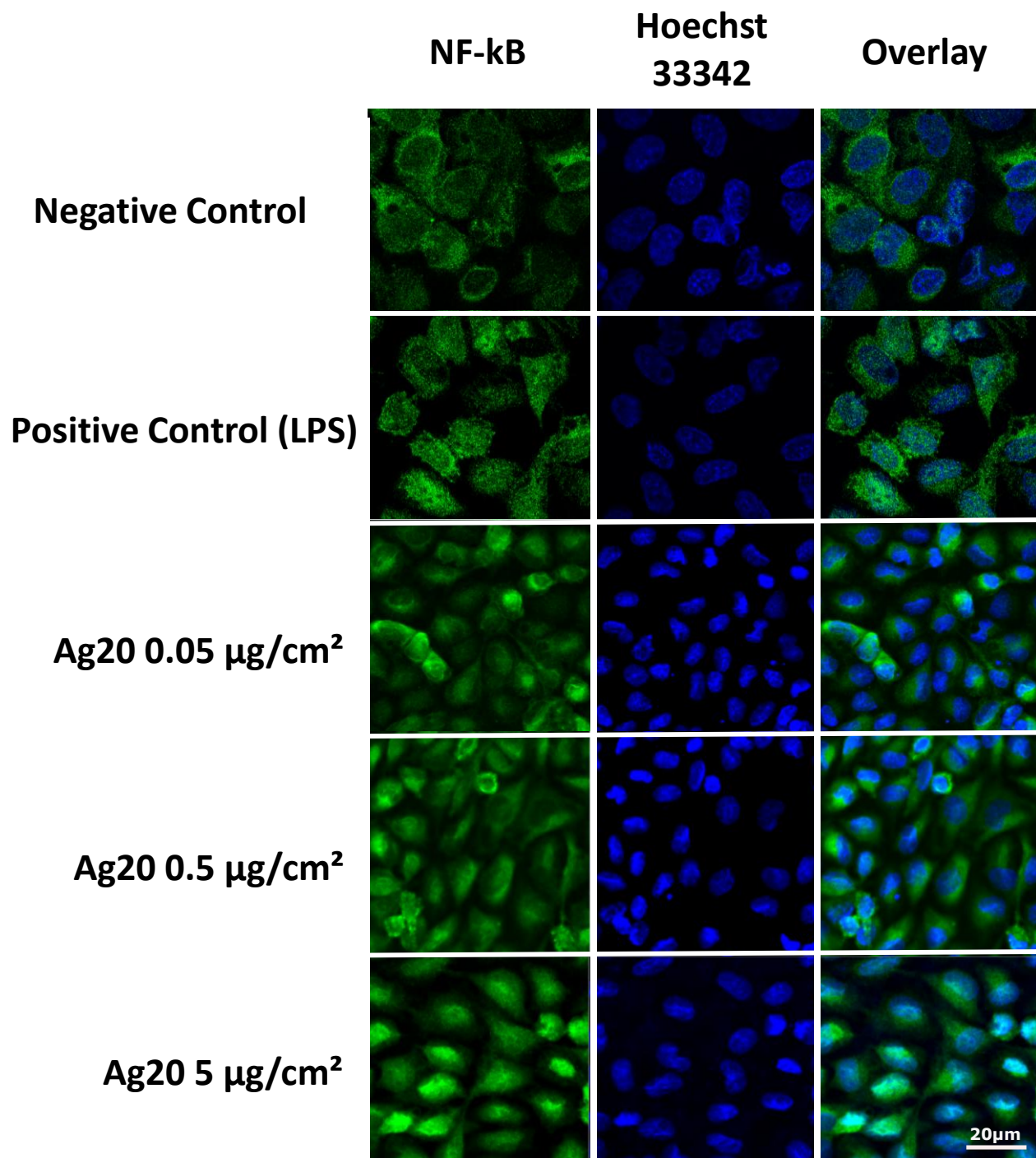

Supplement: Supplementary file 10 — Figure S9. Potential of Ag20 to induce the nuclear translocation of NF-kB in endothelial cells at 4 h post-exposure. The alveolar model was exposed to Ag20 at the three concentrations studied. Cells exposed to vehicle served as Negative Control. Positive Control was exposed to Lipopolysaccharides (LPS) at a concentration of 10 μg/mL for the same period of time. Cells were fixed and stained for NF-kB (green) and nuclei (blue). (PDF 1722 kb) [file 12989_2019_297_MOESM10_ESM.pdf]

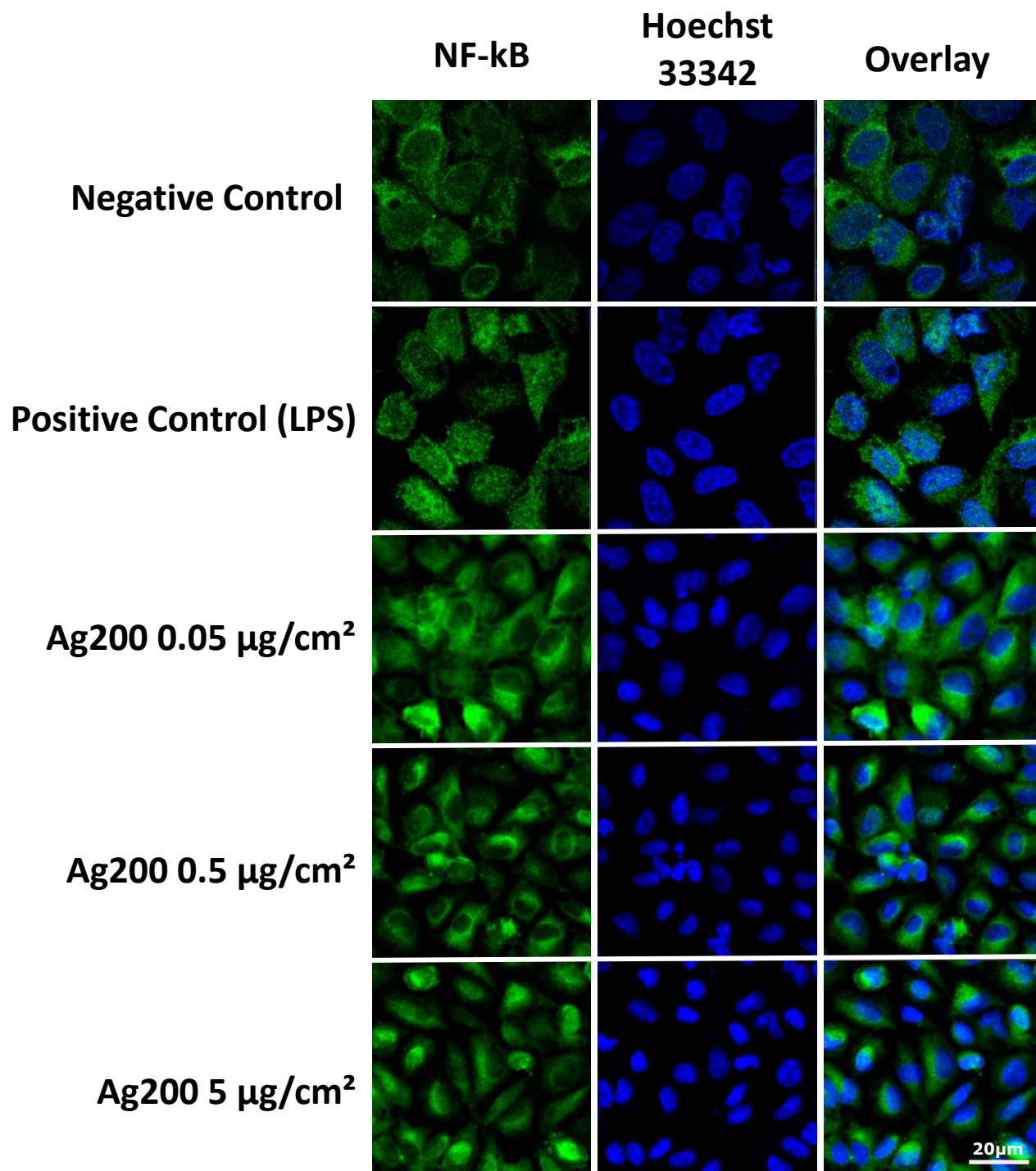

Supplement: Supplementary file 11 — Figure S10. Potential of Ag200 to induce the nuclear translocation of NF-kB in endothelial cells at 4 h post-exposure. The alveolar model was exposed to Ag200 at the three concentrations studied. Cells exposed to vehicle served as Negative Control. Positive Control was exposed to Lipopolysaccharides (LPS) at a concentration of 10 μg/mL for the same period of time. Cells were fixed and stained for NF-kB (green) and nuclei (blue). (PDF 1773 kb) [file 12989_2019_297_MOESM11_ESM.pdf]

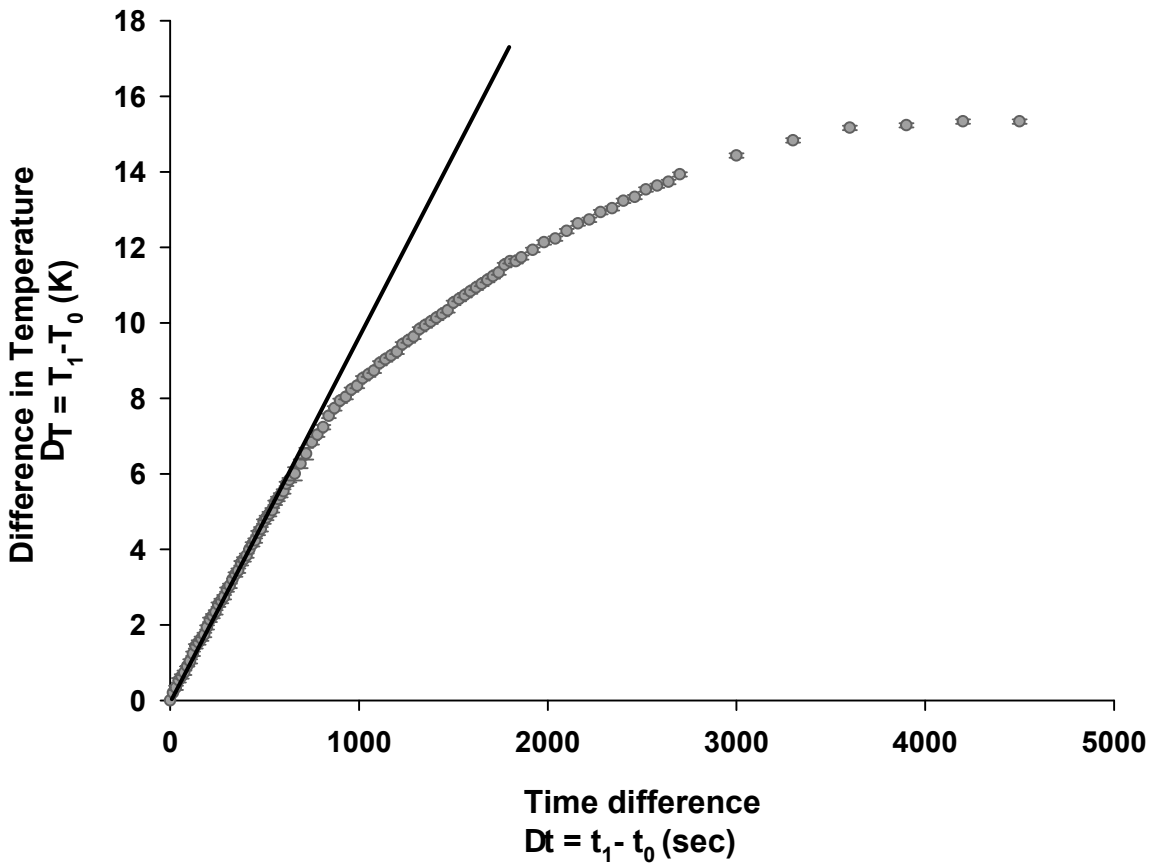

Supplement: Supplementary file 12 — Figure S11. Calorimetric calibration of the UP200S sonicator. 50 mL of ddH2O were sonicated at amplitude of 50% in a continuous mode up to 4800 s and the difference in temperature was measured. The plot representing the difference in temperature (ΔT) as a function of the sonication period (Δt) was constructed, and from the first linear portion, the delivered acoustic power (W = Joule/Second) was calculated. (PDF 26 kb) [file 12989_2019_297_MOESM12_ESM.pdf]

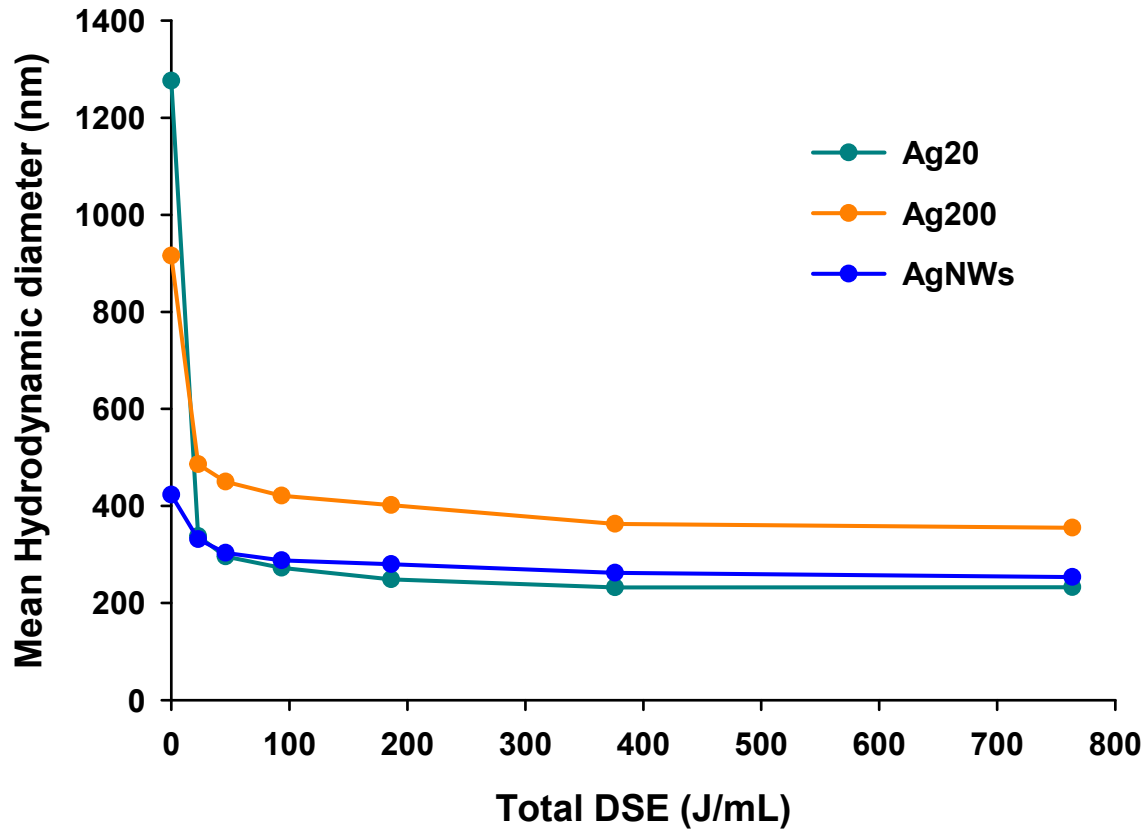

Supplement: Supplementary file 13 — Figure S12. Determination of Critical Delivered Sonication Energy (DSECR) for Ag20, Ag200 and AgNWs. The plot is presenting the mean hydrodynamic diameter as a function of the total delivered sonication energy (DSE). Hydrodynamic diameter was measured by DLS. (PDF 19 kb) [file 12989_2019_297_MOESM13_ESM.pdf]
