# Supplementary material for: In vitro exposure of a 3D-tetraculture representative for the alveolar barrier at the air-liquid interface to silver particles and nanowires
Source: Part Fibre Toxicol. 2019 Apr 2;16:14. doi: 10.1186/s12989-019-0297-1 (PMC6444883; doi:10.1186/s12989-019-0297-1)
Supplement: Supplementary file 2 — Table S1. Intra- and inter-exposure variability calculated based on the deposited quantities of Ag20, Ag200 and AgNWs during three independent exposures (Exp_1, Exp_2 and Exp_3), using the Vitrocell™Cloud System.Table S2. Complete media for mono-cultures and tetra-culture. Table S3. List of primer sequences used for qRT-PCR experiments on the apical and basolateral side of the alveolar model. (DOCX 23 kb) [file 12989_2019_297_MOESM2_ESM.docx]

**Supplemental Material Table S1.** Intra- and inter-exposure variability calculated based on the deposited quantities of Ag20, Ag200 and AgNWs during three independent exposures (Exp_1, Exp_2 and Exp_3), using the Vitrocell™Cloud System.

|  | | **Intra-exposure CV (%)** | | | **Inter-exposure CV (%)** | | |
| --- | --- | --- | --- | --- | --- | --- | --- |
|  | | 50 µg/mL | 500 µg/mL | 5000 µg/mL | 50 µg/mL | 500 µg/mL | 5000 µg/mL |
| **Ag20** | Exp_1 | 26.4 | 10.1 | 13.3 |  |  |  |
|  | Exp_2 | 26.3 | 14.6 | 10.0 | 28.1 | 36.5 | 9.8 |
|  | Exp_3 | 19.6 | 15.8 | 7.6 |  |  |  |
| **Ag200** | Exp_1 | 39.2 | 49.3 | 14.9 |  |  |  |
|  | Exp_2 | 24.6 | 36.1 | 21.2 | 31.9 | 36.5 | 20.5 |
|  | Exp_3 | 33.0 | 27.9 | 24.7 |  |  |  |
| **AgNWs** | Exp_1 | 22.5 | 48.6 | 11.2 |  |  |  |
|  | Exp_2 | 29.3 | 25.3 | 19.1 | 33.0 | 33.7 | 18.7 |
|  | Exp_3 | 35.5 | 23.4 | 21.1 |  |  |  |

**Supplemental Material Table S2.** Complete media for mono-cultures and tetra-culture.

| **Mono-cultures** | | | |
| --- | --- | --- | --- |
| **Cell line** | **Medium** | **Serum** | **Supplements** |
| **A549** | DMEM | 10% (v/v) FBS |  |
| **THP-1** | RPMI-1640 | 10% (v/v) FBS | 25 mM HEPES  50 µM β-mercaptoethanol |
| **HMC-1** | IMDM | 10% (v/v) FBS | 25 mM HEPES  1.2 mM α -thioglycerol |
| **Ea.hy 926** | DMEM | 10% (v/v) FBS | 25 mM HEPES |
| **Tetra-culture** | | | |
| **Condition** | **Medium** | **Serum** | **Supplements** |
| Submerged, growth and proliferation | 75% HEPES-buffered DMEM; 15% RPMI; 10% IMDM | 10% (v/v) FBS | 25 mM HEPES |
| ALI, maintenance and exposure | 75% HEPES-buffered DMEM; 15% RPMI; 10% IMDM | 1% (v/v) FBS | 25 mM HEPES |
| DMEM = Dulbecco’s Modified Eagle’s Medium; RPMI-1640 = Roswell Park Memorial Institute 1640; IMDM = Iscove’s Modified Dulbecco’s Medium; FBS = Fetal Bovine Serum Gold | | | |

**Supplemental Material Table S3.** List of primer sequences used for qRT-PCR experiments on the apical and basolateral side of the alveolar model.

| **Gene** | **Forward Primer** (5’🡪3’) | **Reverse Primer** (5’🡪3’) | **Reference** |
| --- | --- | --- | --- |
| MT-1A | GGATCTCCAACCTCACCGC | GCACTTCTCTGATGCCCCTT |  |
| MT-1B | GGAACTCCAGGCTTGTCTTG | CAGCGGCACTTCTCTGATGA |  |
| MT-2A | AACTGCTCCTGCGCCGC | GCGTTCTTTACATCTGGGAGCG |  |
| GCSF-R | TGGAGCTGAGAACTACCGAA | CCTGAGGGTCTCCAAGAAA |  |
| HMOX-1 | TTCTCCGATGGGTCCTTACACT | GGCATAAAGCCCTACAGCAACT | [26] |
| CASP-7 | CGGTCCTCGTTTGTACCGTC | GGTGGTCTTGATGGATCGCA | [26] |
| SOD-1 | GTGCAGGTCCTCACTTTAAT | CTTTGTCAGCAGTCACATTG | [26] |
| FAS | AGCTTGGTCTAGAGTGAAAA | GAGGCAGAATCATGAGATAT | [26] |
| NF-kB | GCTTGTAGGAAAGGACTGCC | GTTGTTGTTGGTCTGGATGC | [26] |
| IL-6 | GGAGACTTGCCTGGTGAAAA | GTTGGGTCAGGGGTGGTTAT | [26] |
| IL-8 | AAGACATACTCCAAACCTTTCCACC | CAATAATTTCTGTGTTGGCGCA |  |
| COX-2 | TCGATTCTTTGCCCAGCACT | AAAGGCGCAGTTTACGCTGT |  |
| NEF2 | ACATTGAGCAAGTTTGGGAG | TGTGGACTACAGTTACCTAC | [26] |
| NQO1 | GGAGAGTTTGCTTACACTTACGC | TTCTCCAGGCGTTTCTTCCA | [26] |
| ICAM-1 | GCAAGGTGACCGTGAATGT | GCATAAAGCCCTACAGCAAC | [26] |
| GST1 | ACAGTTGTACAAGTTGCAGGATG | TGCCAAAGAGATTGTGCTTG | [26] |
| HSP70 | CCTACTCCGACAACCAACCC | GGTGATCTTGTTGGCCTTGC | [26] |
| HMOX-2 | GGGAAAGGAGACATGCGTAA | CAAGAGTCCAGCAGCTAGGG | [26] |
| E-SELECTIN | ACCTCCACGGAAGCTATGACT | CAGACCCACACATTGTTGACTT | [26] |
| VCAM-1 | CTTAAAATGCCTGGGAAGATGGT | GTCAATGAGACGGAGTCACCAAT | [26] |
| B2M | TGCTGTCTCCATGTTTGATGTATCT | TCTCTGCTCCCCACCTCTAAGT | [26] |
| HPRT-1 | TGACACTGGCAAAACAATGCA | GGTCCTTTTCACCAGCAAGCT | [26] |
| YWHAZ | ACTTTTGGTACATTGTGGCTTCAA | CCGCCAGGACAAACCAGTAT | [26] |
| SDHA | TGGGAACAAGAGGGCATCTG | CCACCACTGCATCAAATTCATG | [26] |
| Unless further mentioned, qRT-PCR primer were designed for the experiments of this study. Abbreviations used: *MT-(1A, 1B, 2A)*- Metallothionein-(1A, 1B, 2A*), GCSF-R* - Granulocyte Colony-Stimulating Factor Receptor, *HMOX-1(2)-* Heme Oxygenase 1(2), *CASP-7* - Caspase 7, *SOD-1 -* Superoxide Dismutase-1*,* *FAS* - Fas Cell Surface Death Receptor, *NF-kB* - Nuclear Factor kappa Light Chain Enhancer of Activated B cells, *IL-6(8)* – Interleukin 6(8), *COX-2* - Cyclooxygenase-2, *NEF2 -* Nuclear factor, Erythroid 2 like 2*,* *NQO1 -* NAD(P)H Quinone Dehydrogenase 1, *ICAM-1* - Intercellular Adhesion Molecule-1, *GST1* - Glutathione S-transferase-1, *HSP70* -Heat Shock Protein 70, *E-SELECTIN* - Endothelial-Leukocyte Edhesion Molecule 1, *VCAM-1* - Vascular Cell Adhesion Molecule-1, *B2M -* Beta-2-Microglobulin, *HPRT-1 -* Hypoxanthine Phosphoribosyltransferase-1, YWHAZ - Tyrosine 3-Monooxygenase/tryptophan 5-Monooxygenase Activation Protein Zeta, SDHA - Succinate Dehydrogenase Complex Flavoprotein Subunit A. | | | |
